# Appropriateness of Antibiotic Prescriptions in Chinese Primary Health Care and the Impact of the COVID-19 Pandemic: A Typically Descriptive and Longitudinal Database Study in Yinchuan City

**DOI:** 10.3389/fphar.2022.861782

**Published:** 2022-04-14

**Authors:** Houyu Zhao, Shengfeng Wang, Ruogu Meng, Guozhen Liu, Jing Hu, Huina Zhang, Shaohua Yan, Siyan Zhan

**Affiliations:** ^1^ Department of Epidemiology and Biostatistics, School of Public Health, Peking University, Beijing, China; ^2^ National Institute of Health Data Science, Peking University, Beijing, China; ^3^ Peking University Health Information Technology, Beijing, China; ^4^ Beijing Hospital of Traditional Chinese Medicine, Capital Medical University, Beijing Institute of Traditional Chinese Medicine, Beijing, China; ^5^ Department of Oncology, Dongfang Hospital Beijing University of Chinese Medicine, Beijing, China; ^6^ Research Center of Clinical Epidemiology, Peking University Third Hospital, Beijing, China; ^7^ Center for Intelligent Public Health, Institute for Artificial Intelligence, Peking University, Beijing, China

**Keywords:** antibiotics, primary health care, COVID-19 pandemic, prescription, outpatient

## Abstract

**Background:** The appropriateness of antibiotic prescriptions in primary care has not been well evaluated in China in recent years. Furthermore, the impact of coronavirus disease 2019 (COVID-19) on antibiotic prescriptions has not yet been investigated in China. We aimed to assess the appropriateness of antibiotic prescriptions and to evaluate the potential association between the COVID-19 pandemic and antibiotic prescriptions in primary care settings of Yinchuan, a city in China.

**Methods:** This study included 155 primary care institutions and 10,192,713 outpatient visits. Outpatient prescriptions were classified as appropriate, potentially appropriate, inappropriate, or not linked to any diagnosis for antibiotic use following a validated evaluation scheme. Interrupted time-series analyses were performed to assess the effects of the COVID-19 pandemic on antibiotic prescriptions in Chinese primary care facilities.

**Results:** During the study period, 1,287,678 (12.6%, 95% confidence interval [12.6–12.7]) of 10,192,713 outpatient visits in primary care resulted in antibiotic prescriptions. Among 1,287,678 antibiotic prescriptions, 653,335 (50.7% [50.6–50.9]) were inappropriate, 463,081 (36.0% [35.8–36.1]) were potentially appropriate, 171,056 (13.3% [13.1–13.5]) were appropriate, and 206 could not be linked to any diagnosis. Furthermore, patient, physician, and institutional factors were associated with inappropriate antibiotic prescriptions; there was an overall decreasing trend in the proportions of inappropriate antibiotic prescriptions, with the highest level in 2017 (67.1% [66.8–67.5]) and the lowest in 2021 (40.8% [40.3–41.3]). A total of 1,416,120 individual antibiotics were prescribed, of which 1,087,630 (76.8%) were broad-spectrum and 777,672 (54.9%) were classified in the World Health Organization’s “Watch” category. In addition, the COVID-19 pandemic was associated with changes of −2.8% (−4.4 to −1.3) in the level and 0.3% (0.2–0.3) in the monthly trend of antibiotic prescription rates, as well as changes of −5.9% (−10.2 to −1.5) in the level and 1.3% (1.0–1.6) in the monthly trend of the proportions of inappropriate antibiotic prescriptions.

**Conclusion:** More than half of the antibiotic prescriptions were inappropriate during the study period in primary care in Yinchuan. The COVID-19 pandemic may be associated with a decrease in the overall and inappropriate use of antibiotics in primary care settings in China.

## Introduction

Inappropriate use of antibiotics is a major driver of antimicrobial resistance (AMR) (World Health Organization, 2015; Holmes et al., 2016), which increases morbidity and mortality and causes substantial economic losses (World Health Organization, 2015; O’Neill, 2016). Worldwide, the majority of antibiotics used in humans are prescribed in primary care settings (Dyar et al., 2016; Peñalva et al., 2020). However, up to or even more than a half of these antibiotic prescriptions are inappropriate (Wang et al., 2014; Dyar et al., 2016; Peñalva et al., 2020). Hence, optimizing the use of antibiotics in primary care settings is essential to curb AMR (World Health Organization, 2015; Dyar et al., 2016). To achieve this goal, information on the pattern and appropriateness of antibiotic use is helpful in informing strategies and policies (World Health Organization, 2018; Sulis et al., 2020a). The increasing consumption of antibiotics and high AMR rates are particular issues in low- and middle-income countries (LMICs) (Holmes et al., 2016; Klein et al., 2018; Sulis et al., 2020a; Miller et al., 2021), where more than 50% of patients in primary care settings receive antibiotic prescriptions (Sulis et al., 2020a). However, antibiotic use in primary care settings has been poorly evaluated in LMICs, where the available studies often suffer from serious limitations, including methodological issues, a non-comprehensive evaluation framework, lack of details of patient features, and an insufficient number of health institutions (Sulis et al., 2020a).

China is the largest LIMC and second largest consumer of antibiotics worldwide (Van Boeckel et al., 2014; Klein et al., 2018). Surveillance in China has also documented a high prevalence of drug-resistant bacteria for a variety of commonly used antibiotics (Xiao et al., 2011; China Antimicrobial Resistance Surveilance System, 2020), making the country one of the largest contributors to AMR (Xiao et al., 2011; Frost et al., 2019). The primary care system in China provides more than 52% of outpatient care services (over four billion visits) (National Health Commission of the People’s Republic of China, 2021), during which a substantial number of antibiotics are prescribed (Yin et al., 2013; Wang et al., 2014). A previous study showed that over 50% of antibiotic prescriptions in Chinese secondary and tertiary hospitals might be inappropriate (Zhao et al., 2021a). However, the appropriateness of antibiotic prescriptions in Chinese primary care facilities has not been well assessed. Several studies have mainly focused on urban community health service centers (CHSCs) and rural township hospitals (THs) (Wang et al., 2014; Chang et al., 2019; Taxifulati et al., 2021), leaving a gap in the appropriateness of antibiotic prescriptions at their subordinate community health service stations (CHSSs) and village clinics (VCs), which are the most basic medical institutions in Chinese primary care settings (Li et al., 2017). Furthermore, these studies have been conducted through manual prescription reviews (Wang et al., 2014; Chang et al., 2019; Taxifulati et al., 2021), which are highly dependent on the pharmacist’s or physician’s level of experience (Zhao et al., 2021a). In addition, several studies have indicated that the coronavirus disease 2019 (COVID-19) pandemic could have significant effects on antibiotic prescriptions (Buehrle et al., 2020; King et al., 2020; Lepak et al., 2021), whereas this evidence is scarce in China. Detailed analyses of antibiotic use throughout this pandemic will help to understand the impact of COVID-19 and inform strategies for controlling AMR (Knight et al., 2021; Miller et al., 2021). In this study, we aimed to use a well-established evaluation framework (Fleming-Dutra et al., 2016; Chua et al., 2019; Zhao et al., 2020; Zhao et al., 2021a) to assess the appropriateness of antibiotic prescriptions in primary care settings in a Chinese city, as well as evaluate the impacts of the COVID-19 pandemic on the antibiotic prescriptions.

## Materials and Methods

### Setting, Data Source, and Participants

The primary care system in China consists of urban and rural components: CHSCs and their satellite station sites (CHSSs) in urban areas, and THs and their outreach VCs in rural areas (Li et al., 2017). These institutions are primarily responsible for providing basic clinical care and public health services (Li et al., 2017). In China, a licensed doctor registered with local health administrative departments can be granted the authority to prescribe antibiotics after passing an examination on antibiotics (Ministry of Health of the People’s Republic of China, 2007; Ministry of Health of the People’s Republic of China, 2012). Doctors in primary care can be licensed in several ways: 1) completion of a college medical program and passing the National Practicing Doctor Examination; 2) completion of a junior college medical training program and passing the National Practicing Assistant Doctor Examination; and 3) village doctors who have practiced continuously in VCs for more than 20 years or received technical school education are permitted to work only in VCs with a village doctor certificate in lieu of a regular license (State Council of the People’s Republic of China, 2003; Central People’s Government of the People’s Republic of China, 2005; Li et al., 2017). However, all doctors in primary care institutions can only prescribe antibiotics that are included on the Essential Medicines List (Ministry of Health of the People’s Republic of China, 2012).

This study was restricted to primary care in Yinchuan, the capital city of the Ningxia Autonomous Region located in northwest China. At the end of 2019, Yinchuan had a population of 2 million across a geographic area of 9,025 km^2^. The gross domestic product in 2019 was 189.7 billion, among which the tertiary industry accounted for 52.8% in this city. The gross domestic product per capita was 83,492 Yuan, ranking in the middle range of all Chinese cities (Department of Urban Surveys, National Bureau of Statistics of China, 2020). In 2016, the Yinchuan Municipal Government established the Yinchuan Primary Healthcare Database to gradually integrate all medical records from all primary care institutions in the city. By 2020, 182 institutions were recruited and required to upload all medical records to the data center every 6 months. All data were stored in the Oracle database on a physically isolated server. All primary care institutions used the same hospital information system, and diagnoses were coded in accordance with the International Classification of Diseases 10th Revision (ICD-10) or the national codes of diseases and ZHENG of traditional Chinese medicine (TCM) when the physicians wrote prescriptions. Antibiotics for systematic use were coded using the Anatomical Therapeutic Chemical (ATC) classification system according to the generic name and route of administration. Drug prescriptions and diagnostic records of the same visit were linked through a unique identifier consisting of the institution code, patient identification number, and date of visit. All visits from 1 June 2017, to 31 July 2021 were included.

In this study, 27 primary care institutions with 6,124 outpatient visits (0.06%) were excluded because less than 10 months of data were uploaded; the remaining 155 institutions (51 CHSCs/THs and 104 CHSSs/VCs) were included in the final analyses.

### Definition of Antibiotics and Outpatient Visits

We assessed antibiotics for systemic use according to ATC code J01. Three other antibiotics, including metronidazole, tinidazole, and ornidazole, were also included because these antibiotics are mainly used to treat anaerobic bacterial infections in China (Bao et al., 2016). Supplementary Table S1 provides a full list of the antibiotics used in this study. Based on previous studies (Zhao et al., 2021a; Zhao et al., 2021b), second- to fourth-generation cephalosporins, fluoroquinolones, macrolides, combinations of penicillins, and aminoglycosides were classified as broad-spectrum antibacterial agents. Other antibiotics were classified as narrow-spectrum antibiotics, such as β-lactamase-sensitive penicillins and first-generation cephalosporins. We also classified antibiotics according to the WHO Access, Watch, and Reserve (AWaRe) categories (World Health Organization, 2020). Only one type of antibiotic, fosfomycin, was classified as Reserve. Four types of antibiotics (cefathiamidine, cefoperazone/tazobactam, cefoperazone/sulbactam, and etimicin) that were not included in any of the AWaRe groups were defined as unclassified.

In this study, multiple prescriptions of drugs and diagnoses from the same patient on the same day in the same primary care institution were treated as one visit.

### Diagnosis Classification

China gives equal attention and weight to TCM and allopathic medicine in primary care; thus, TCM care is widely provided in primary care institutions, often jointly with allopathic medical care (Li et al., 2017). In this study, TCM diagnoses accounted for approximately 13% of all diagnostic records. We first classified all allopathic medicine diagnoses, which were coded using ICD-10, into three tiers following the approach applied in previous studies (Fleming-Dutra et al., 2016; Chua et al., 2019; Hashimoto et al., 2020; Zhao et al., 2020; Zhao et al., 2021a; Zhao et al., 2021b): 1) “tier 1” if the condition almost always justifies antibiotics, 2) “tier 2” if the condition only sometimes justifies antibiotics, and 3) “tier 3” if the condition almost never justifies antibiotics. Furthermore, diagnoses were classified into 30 different categories as in our previous studies (Zhao et al., 2021a; Zhao et al., 2021b). Details of the classification framework based on the ICD-10 have been published elsewhere (Zhao et al., 2020; Zhao et al., 2021a) and are also given in Supplementary Table S2. TCM diagnoses of antibiotic prescriptions were also classified into the three tiers as described above by two TCM physicians independently. Inconsistent classifications were reviewed by a third expert. After all diagnoses were classified, a single diagnosis category was assigned to each antibiotic prescription in the following order of priority when multiple diagnoses of the same tier existed in a visit: tier 1 diagnosis, followed by tier 2 diagnosis, and tier 3 diagnosis (Fleming-Dutra et al., 2016; Chua et al., 2019; Zhao et al., 2020; Zhao et al., 2021a). In addition, priority was given to allopathic medical diagnoses when TCM diagnoses jointly existed in the same tier. This tier-method was conservative because only one diagnosis justifying antibiotics is required to classify the visit as appropriate or potentially appropriate (Olesen et al., 2018; Zhao et al., 2021a). However, when describing the antibiotic prescription rates for visits with different diagnostic categories, all TCM diagnoses were classified into a single category.

### Antibiotic Prescription Rates and Appropriateness of Antibiotic Prescriptions

Antibiotic prescription rates were calculated as the percentage of outpatient visits that ended with antibiotic prescriptions for different diagnostic categories in urban and rural primary care. Antibiotic prescriptions were classified into one of the four mutually exclusive categories as applied in previous studies (Chua et al., 2019; Zhao et al., 2021a): “appropriate” if a tier 1 diagnosis was assigned, “potentially appropriate” if a tier 2 diagnosis and no tier 1 diagnosis was assigned, “inappropriate” if only tier 3 diagnosis was assigned, and “not-linked to any diagnosis” if no visit-level diagnosis was linked to the prescription. The proportions of visits ending with antibiotic prescriptions in all appropriateness categories within different subgroups were calculated.

Antibiotic prescriptions were examined overall and by area type (urban and rural), institution level (CHSCs/THs and CHSSs/VCs), patient gender, age group (<6, 6–17, 18–45, 46–64, and ≥65 years), payment type (by insurance or full out-of-pocket), and year of outpatient visit (2017–2021), as well as physician characteristics, including age (<30, 30–39, 40–49, 50–59, and ≥60), gender, and education level. Antibiotic prescription patterns were identified by calculating the proportions of different forth-level ATC categories, broad-spectrum agents, and WHO AWaRe categories.

### Statistical Analysis

Descriptive statistics of the outcome measures for the entire study group, as well as for different diagnostic categories and subgroups, were calculated. The Clopper-Pearson exact method was used to calculate the 95% confidence intervals (CIs) of the antibiotic prescription rates. The simultaneous 95% CIs for multinomial proportions of antibiotic prescriptions for all four appropriateness categories were estimated using the Goodman method (May and Johnson, 1997). A multi-variate binary logistic regression with random intercept for each primary care institution was performed to assess the potential influencing factors of inappropriate antibiotic prescriptions. Categories except for inappropriate were combined into a single category, and odds ratios (ORs) with 95% CIs were reported for inappropriate antibiotic prescriptions.

Two sensitivity analyses were performed for the appropriateness classification of TCM diagnoses. First, all TCM diagnoses were classified as tier 2 diagnoses. Second, all TCM diagnoses were classified as tier 3 diagnoses. The appropriateness category was reassigned to each antibiotic prescription using the tier-fashion method mentioned above and the proportion of each appropriateness category was recalculated. We also calculated the diagnosis category-standardized antibiotic prescription rates using the disease spectrum of the whole population during the whole study period as the standard population to check whether the differences between subgroups were due to changes in the disease spectrum of visits.

Interrupted time series analyses using segmented linear regressions were conducted to assess the impact of the COVID-19 pandemic on antibiotic prescription rates and proportions of inappropriate antibiotic prescriptions. Given the delay of the intervention effect, March 2020, when the WHO declared COVID-19 a pandemic, was set as the time when the pandemic began to have an impact, according to descriptive trend analyses and previous studies (Buehrle et al., 2020; Agca et al., 2021; Lepak et al., 2021).

Interrupted time series analyses regression models were fitted for outcome measures (antibiotic prescription rates or proportions of inappropriate antibiotic prescriptions) with the following equation:

$$Y_{t}=\beta_{0}+\beta_{1}t_{0}+\beta_{2}Intv\_Covid19_{t}+\beta_{3}t_{aft\_Covid\_19}+\epsilon_{t}$$

In this model, $Y_{t}$ is the outcome variable measured in each month; $t_{0}$ is the time measured in months since June 2017; $Intv\_Covid19_{t}$ is a dummy indicator representing the impact of the COVID-19 pandemic with a value of 0 before March 2020 and 1 thereafter; $\;t_{aft\_Covid19}$ is the time after the pandemic begins to have an impact. In this model specification, $\beta_{0}$ represents the starting level of the outcome variable. *β*_1_ is the baseline slope of the outcome variable until the COVID-19 pandemic period. *β*_2_ represents the change in the level of the outcome that occurred immediately following the pandemic and *β*_3_ represents the slope change in the outcomes after the COVID-19 pandemic. The SAS software X12 procedure was used to identify seasonal variations in antibiotic prescriptions. This method is an adaptation of the United States Bureau of the Census X-12-Auto-Regressive Integrated Moving Average model, which produces a seasonally adjusted time-series (Jackson and Leonard, 2000). We conducted the Cumby-Huizinga test and calculated the Durbin-Watson statistic to test for serial autocorrelation of the error terms. The Newey-West method was used to handle autocorrelation, in addition to possible heteroskedasticity. Subgroup analyses were performed for the different area types and facility levels.

Data extraction and diagnosis classification were performed using Oracle 11gR2 (Oracle Corp., Redwood Shores, CA, United States). Statistical analyses were performed using SAS 9.4 (SAS Institute Inc., Cary, NC, United States) and Stata 16.0 (StataCorp, College Station, TX, United States).

### Ethics Statement

This study was approved by the Ethical Review Board of Peking University Health Science Center (approval number: IRB00001052-18013-Exempt). Informed consent was not required because we used anonymized routine data.

## Results

### Basic Characteristics

A total of 10,192,713 outpatient visits from 155 primary care institutions were included in this study. Among these visits, 6,240,795 (61.2%) occurred in urban areas and 5,376,777 (52.8%) occurred in CHSCs/THs. In addition, 1,358,368 (13.4%) visits were made by children, 4,684,654 (46.0%) were made by men, and 8,059,694 (79.1%) were paid with insurance. Of all visits, 56.6% (5,763,780) were served by physicians aged 40 years and older, 5,707,639 (56.0%) by female physicians, and 7,695,231 (75.5%) by physicians with an education level of high school and below (Table 1).

**TABLE 1:** Basic characteristics of outpatient visits in primary care setting in Yinchuan City.

	Urban, *n* (%)	Rural, *n* (%)	All Regions, *n* (%)
Overall	6,240,795 (61.2)	3,951,918 (38.8)	10,192,713 (100.0)
Type of primary care			
CHSCs/THs	1,697,017 (27.2)	3,679,760 (93.1)	5,376,777 (52.8)
CHSSs/VCs	4,543,778 (72.8)	272,158 (6.9)	4,815,936 (47.2)
Patient’s age, years			
<6	498,851 (8.0)	220,748 (5.6)	719,599 (7.1)
6–17	311,746 (5.0)	327,023 (8.3)	638,769 (6.3)
18–44	1,209,478 (19.4)	1,095,625 (27.7)	2,305,103 (22.6)
45–64	2,273,517 (36.4)	1,455,837 (36.8)	3,729,354 (36.6)
≥65	1,947,203 (31.2)	852,685 (21.6)	2,799,888 (27.5)
Patient’s gender			
Male	2,851,669 (45.7)	1,832,985 (46.4)	4,684,654 (46.0)
Female	3,389,126 (54.3)	2,118,933 (53.6)	5,508,059 (54.0)
Payment type			
Insurance	4,966,269 (79.6)	3,093,425 (78.3)	8,059,694 (79.1)
Full out-of-pocket	1,274,526 (20.4)	858,493 (21.7)	2,133,019 (20.9)
Year of visit			
2017	501,525 (8.0)	482,008 (12.2)	983,533 (9.6)
2018	1,037,463 (16.6)	1,072,439 (27.1)	2,109,902 (20.7)
2019	1,649,256 (26.4)	983,005 (24.9)	2,632,261 (25.8)
2020	1,811,191 (29.0)	925,029 (23.4)	2,736,220 (26.8)
2021	1,241,360 (19.9)	489,437 (12.4)	1,730,797 (17.0)
Physician’s age, years			
<30	330,470 (5.3)	292,232 (7.4)	622,702 (6.1)
30–39	2,157,095 (34.6)	1,649,136 (41.7)	3,806,231 (37.3)
40–49	1,716,438 (27.5)	1,234,778 (31.2)	2,951,216 (29.0)
50–59	1,367,603 (21.9)	538,717 (13.6)	1,906,320 (18.7)
≥60	669,189 (10.7)	237,055 (6.0)	906,244 (8.9)
Physician gender			
Male	2,241,784 (35.9)	2,243,290 (56.8)	4,485,074 (44.0)
Female	3,999,011 (64.1)	1,708,628 (43.2)	5,707,639 (56.0)
Physician’s education level			
Bachelor degree or above	1,466,171 (23.5)	1,031,311 (26.1)	2,497,482 (24.5)
High school and below	4,774,624 (76.5)	2,920,607 (73.9)	7,695,231 (75.5)

CHSCs, City community health service centers; THs, Township hospitals; CHSSs, community health service stations; VCs, Village clinics.

### Antibiotic Prescription Rate

**FIGURE 1:**
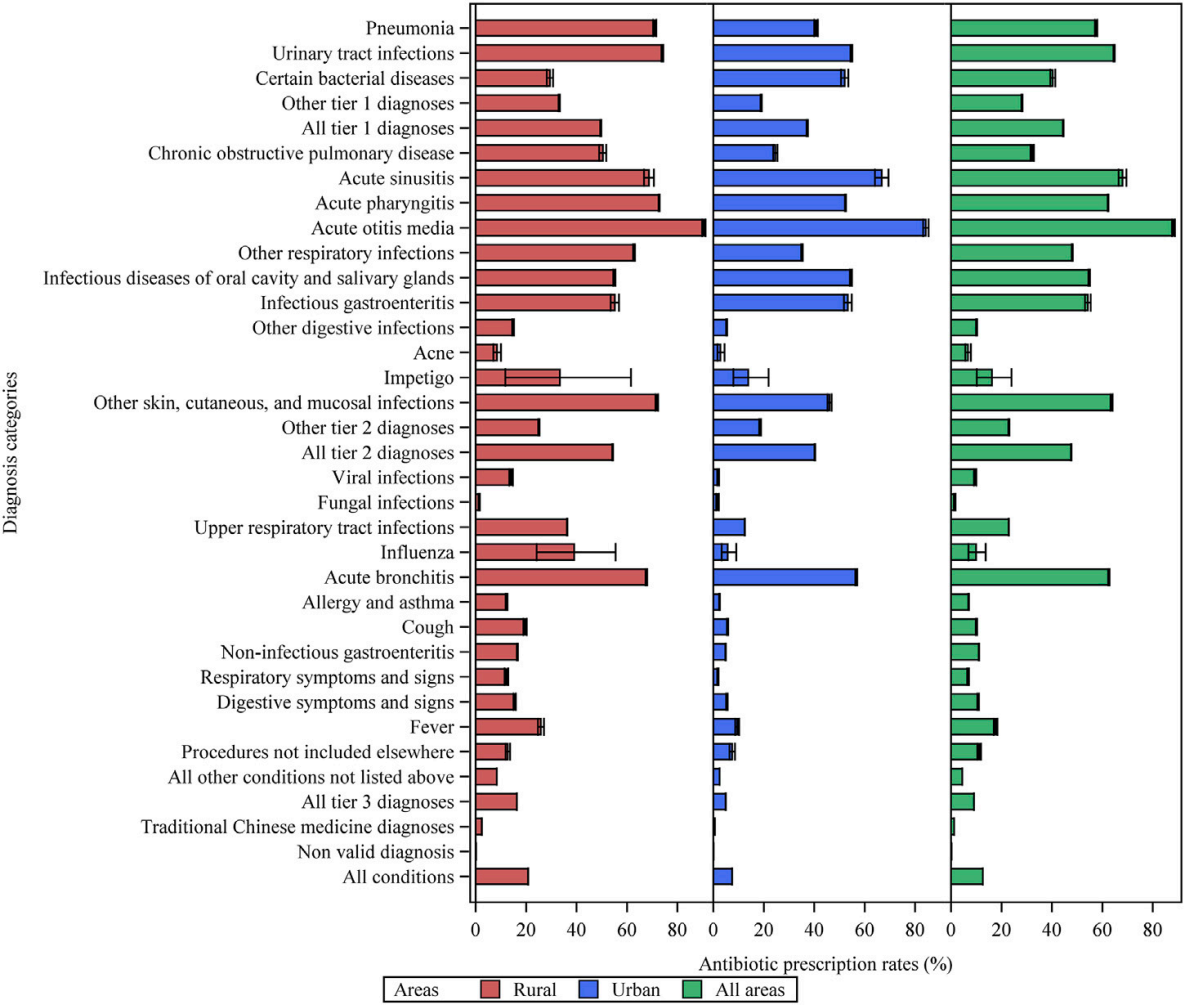
Antibiotic prescription rates for various diagnostic categories of patient visits in primary care settings in Yinchuan City. Other tier 1 diagnoses = other tier 1 bacterial infections. Other respiratory infections = other infectious diseases of the respiratory system categorized in tier 2. Other tier 2 diagnoses = other tier 2 infectious diseases for which an antibiotic may be indicated.

Overall, 12.6% (95% CI: 12.6–12.7, 1,287,678 visits) of the 10,192,713 outpatient visits resulted in antibiotic prescriptions. Antibiotic prescription rates were 7.4% (7.4–7.5) and 20.8% (20.8–20.9) in urban and rural primary care institutions, respectively (Figure 1 and Supplementary Table S3). Among the visits for tier 1 diagnoses, 84,716 (64.7% [64.4–64.9]) of 131,032 visits for urinary tract infections and 23,001 (57.5% [57.0–58.0]) of 40,025 visits for pneumonia were associated with antibiotic prescriptions. As for tier 2 diagnoses, acute otitis media and acute sinusitis were the top two conditions with the highest antibiotic prescription rates, which were 88.2% ([87.6–88.8], 11,003 out of 12,472 visits), and 68.0% ([66.4–69.6], 2,323 out of 3,416 visits), respectively. Antibiotic prescriptions were even prevalent in tier 3 conditions. For patients with acute bronchitis, viral upper respiratory tract infections, unspecific fever, and influenza, antibiotic prescription rates were 62.5% ([62.3–62.8], 107,460 out of 171,855 visits), 22.9% ([22.8–23.0], 273,888 out of 1,196,567 visits), 17.7% ([17.0–18.4]), 1,981 out of 11,216 visits), and 9.9% ([6.9–13.7] 32 out of 323 visits), respectively.

Antibiotic prescription rates before and after standardization for the various subgroups are shown in Supplementary Table S4. For CHSCs/THs, 927,910 of 5,376,777 (17.3% [17.2–17.3]) visits were associated with antibiotic prescriptions, obviously higher than that for CHSSs/VCs, which was 7.5% ([7.4–7.5], 359,768 out of 4,815,936 visits). After standardization, the differences between the different subgroups decreased. For instance, the standardized prescription rates in CHSCs/THs and CHSSs/VCs were 15.8% (15.7–15.8) and 9.0% (9.0–9.1), respectively, indicating that the difference in the diagnostic spectrum might be an important reason for the different antibiotic prescribing among various subgroups.

### Inappropriate Antibiotic Prescriptions

Among all 1,287,678 visits that resulted in antibiotic prescriptions, 653,335 (50.7% [50.6–50.9]) were considered inappropriate, 171,056 (13.3% [13.1–13.5]) were appropriate, 463,081 (36.0% [35.8–36.1]) were potentially appropriate, and 206 (0.02% [0.0–3.0]) visits could not be linked to valid diagnostic records (Table 2). The antibiotic prescriptions tended to be more appropriate in urban primary care institutions and in CHSCs/THs, where the estimated proportions of inappropriate antibiotic prescriptions were 47.1% ([46.8–47.4], 218,706 out of 464,283 visits; OR 0.70 [0.52–0.94]) and 50.6% ([50.4–50.8], 469,646 out of 927,910 visits; OR 0.65 [0.48–0.87]), whereas these proportions were 52.8% ([52.6–53.0], 434,629 out of 823,395 visits), and 51.1% ([50.8–51.3], 183,689 out of 359,768 visits) in rural primary care institutions and CHSSs/VCs, respectively. Children aged <6 years had the highest proportion of inappropriate antibiotic prescriptions at 62.2% ([61.7–62.8], 54,166 out of 87,018 visits), which was 1.56 (1.53–1.58) times that in adults aged 18–44 years. In addition, 53.8% ([53.5–54.0], 220,716 out of 410,571 visits) of the antibiotics prescribed by physicians aged 40–49 years were considered inappropriate, ranking first among physicians of all age groups. Furthermore, 51.6% ([51.5–51.8], 497,712 out of 963,974 visits) of antibiotic prescriptions by physicians with an education level of high school and below were inappropriate, higher than that of physicians with a bachelor’s degree or above. In addition, there was a decreasing trend in the proportion of inappropriate antibiotic prescriptions, with the highest proportion (67.1%, [66.8–67.5]) in 2017 and the lowest proportions in 2020 (40.8% [40.4–41.2]; OR 0.32, [0.32–0.32]) and 2021 (40.8% [40.3–41.3]; OR 0.31, [0.31–0.32]).

**TABLE 2:** Proportion of antibiotic prescriptions in each appropriateness category for various subgroups.^a^

	Appropriate antibiotic use		Potentially appropriate antibiotic use		Inappropriate antibiotic use		OR (95%CI) of inappropriate prescribing
	No. of prescriptions	Proportion, % (95% CI)	No. of prescriptions	Proportion, % (95% CI)	No. of prescriptions	Proportion, % (95% CI)	
Overall	171,056	13.3 (13.1–13.5)	463,081	36.0 (35.8–36.1)	653,335	50.7 (50.6–50.9)	—
Area type							
Urban	60,063	12.9 (12.6–13.3)	185,462	39.9 (39.7–40.2)	218,706	47.1 (46.8–47.4)	0.70 (0.52–0.94)
Rural	110,993	13.5 (13.2–13.7)	277,619	33.7 (33.5–33.9)	434,629	52.8 (52.6–53.0)	1
Type of primary care							
CHSCs/THs	132,065	14.2 (14.0–14.5)	326,032	35.1 (34.9–35.3)	469,646	50.6 (50.4–50.8)	0.65 (0.48–0.87)
CHSSs/VCs	38,991	10.8 (10.5–11.2)	137,049	38.1 (37.8–38.4)	183,689	51.1 (50.8–51.3)	1
Patient’s age, years							
<6	5,452	6.3 (5.5–7.1)	27,383	31.5 (30.8–32.2)	54,166	62.2 (61.7–62.8)	1.56 (1.53–1.58)
6–17	5,803	4.4 (3.8–5.1)	48,207	36.7 (36.2–37.3)	77,186	58.8 (58.4–59.3)	1.33 (1.31–1.35)
18–44	49,405	13.5 (13.1–13.9)	129,052	35.2 (34.9–35.6)	187,838	51.3 (51.0–51.6)	1
45–64	66,021	14.9 (14.6–15.3)	160,505	36.2 (35.9–36.5)	216,324	48.8 (48.6–49.1)	0.99 (0.98–1.00)
≥65	44,375	17.1 (16.6–17.5)	97,934	37.6 (37.3–38.0)	117,821	45.3 (44.9–45.6)	0.93 (0.92–0.94)
Patient’s gender							
Male	63,477	10.3 (10.0–10.7)	228,911	37.3 (37.1–37.6)	320,963	52.3 (52.1–52.5)	1
Female	107,579	16.0 (15.7–16.2)	234,170	34.7 (34.5–35.0)	332,372	49.3 (49.1–49.5)	0.91 (0.90–0.92)
Payment type							
Insurance	142,664	13.4 (13.2–13.7)	385,558	36.3 (36.1–36.5)	534,120	50.3 (50.1–50.4)	1
Full out-of-pocket	28,392	12.6 (12.1–13.1)	77,523	34.4 (34.0–34.9)	119,215	52.9 (52.6–53.3)	0.93 (0.92–0.94)
Year of visit							
2017	13,858	7.8 (7.2–8.4)	44,572	25.1 (24.5–25.6)	119,437	67.1 (66.8–67.5)	1
2018	36,461	11.3 (10.9–11.7)	100,827	31.2 (30.9–31.6)	185,687	57.5 (57.2–57.8)	0.66 (0.65–0.67)
2019	46,225	13.4 (13.0–13.8)	131,028	37.9 (37.6–38.3)	168,186	48.7 (48.4–49.0)	0.45 (0.44–0.46)
2020	46,444	16.8 (16.3–17.2)	117,498	42.4 (42.0–42.8)	113,075	40.8 (40.4–41.2)	0.32 (0.32–0.32)
2021	28,068	17.1 (16.5–17.7)	69,156	42.1 (41.7–42.6)	66,950	40.8 (40.3–41.3)	0.31 (0.31–0.32)
Physician’s age, years							
<30	11,106	14.4 (13.6–15.3)	27,852	36.2 (35.5–36.9)	38,031	49.4 (48.8–50.0)	1
30–39	68,722	14.4 (14.0–14.7)	175,024	36.6 (36.3–36.9)	234,823	49.1 (48.8–49.3)	0.93 (0.92–0.95)
40–49	53,954	13.1 (12.8–13.5)	135,860	33.1 (32.8–33.4)	220,716	53.8 (53.5–54.0)	1.03 (1.01–1.05)
50–59	26,690	11.0 (10.5–11.5)	92,007	37.8 (37.4–38.2)	124,536	51.2 (50.8–51.5)	1.08 (1.05–1.10)
≥60	10,584	13.5 (12.7–14.4)	32,338	41.4 (40.7–42.1)	35,229	45.1 (44.4–45.7)	0.94 (0.91–0.96)
Physician gender							
Male	77,278	11.3 (11.0–11.6)	238,262	34.8 (34.5–35.0)	369,385	53.9 (53.7–54.1)	1
Female	93,778	15.6 (15.3–15.9)	224,819	37.3 (37.1–37.6)	283,950	47.1 (46.9–47.4)	0.87 (0.86–0.88)
Physician’s education level							
Bachelor degree or above	44,469	13.7 (13.3–14.2)	123,510	38.2 (37.8–38.5)	155,623	48.1 (47.8–48.4)	1.02 (1.01–1.04)
High school and below	126,587	13.1 (12.9–13.4)	339,571	35.2 (35.0–35.4)	497,712	51.6 (51.5–51.8)	1

CHSCs, City community health service centers; THs, Township hospitals; CHSSs, community health service stations; VCs, Village clinics.

a 206 (0.02%) antibiotic prescriptions were not linked to any visit level diagnosis and were not presented in this table.

The sensitivity analyses (Supplementary Table S5) showed that the results of inappropriate antibiotic prescriptions were robust for classifications of TCM diagnoses, for which the proportions of inappropriate antibiotic prescriptions were 48.6% ([48.5–48.8], 626,369 out of 1,287,678 visits) when all TCM diagnoses were classified as tier 2 and 53.9% ([53.8–54.0], 694,064 out of 1,287,678 visits) when all TCM diagnoses were classified as tier 3 diagnoses. Supplementary Table S6 provides the estimates of inappropriate antibiotic prescriptions for various subgroups after standardization by disease spectrum, and presents different estimates from those without standardization, indicating that the differences in inappropriate antibiotic prescriptions between different subgroups might be because of the discrepancy in disease spectrums.

### Patterns of Antibiotic Prescriptions

**FIGURE 2:**
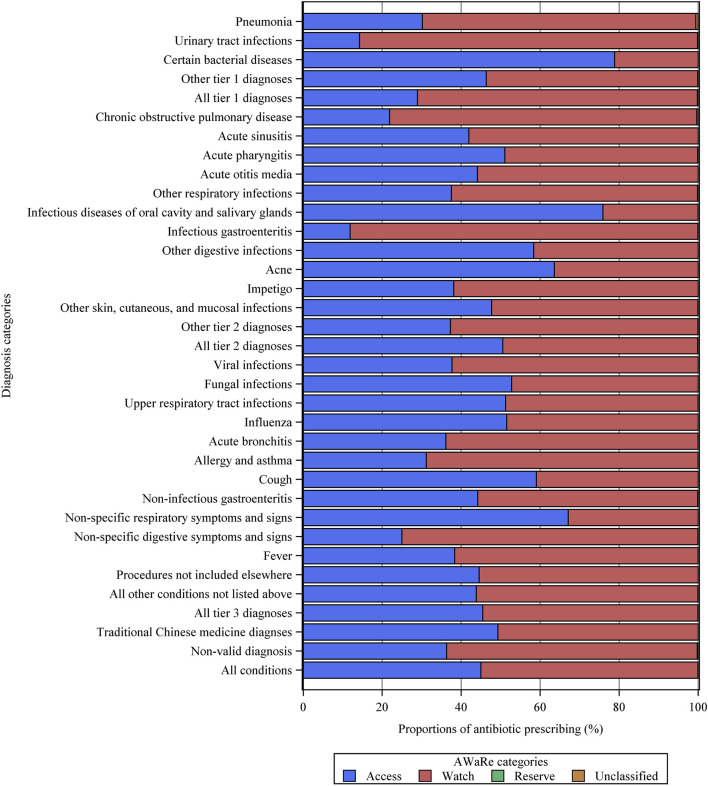
Proportions of different AWaRe categories of antibiotics for various diagnostic categories. Other tier 1 diagnoses = other tier 1 bacterial infections. Other respiratory infections = other infectious diseases of the respiratory system categorized in tier 2. Other tier 2 diagnoses = other tier 2 infectious diseases for which an antibiotic may be indicated.

**FIGURE 3:**
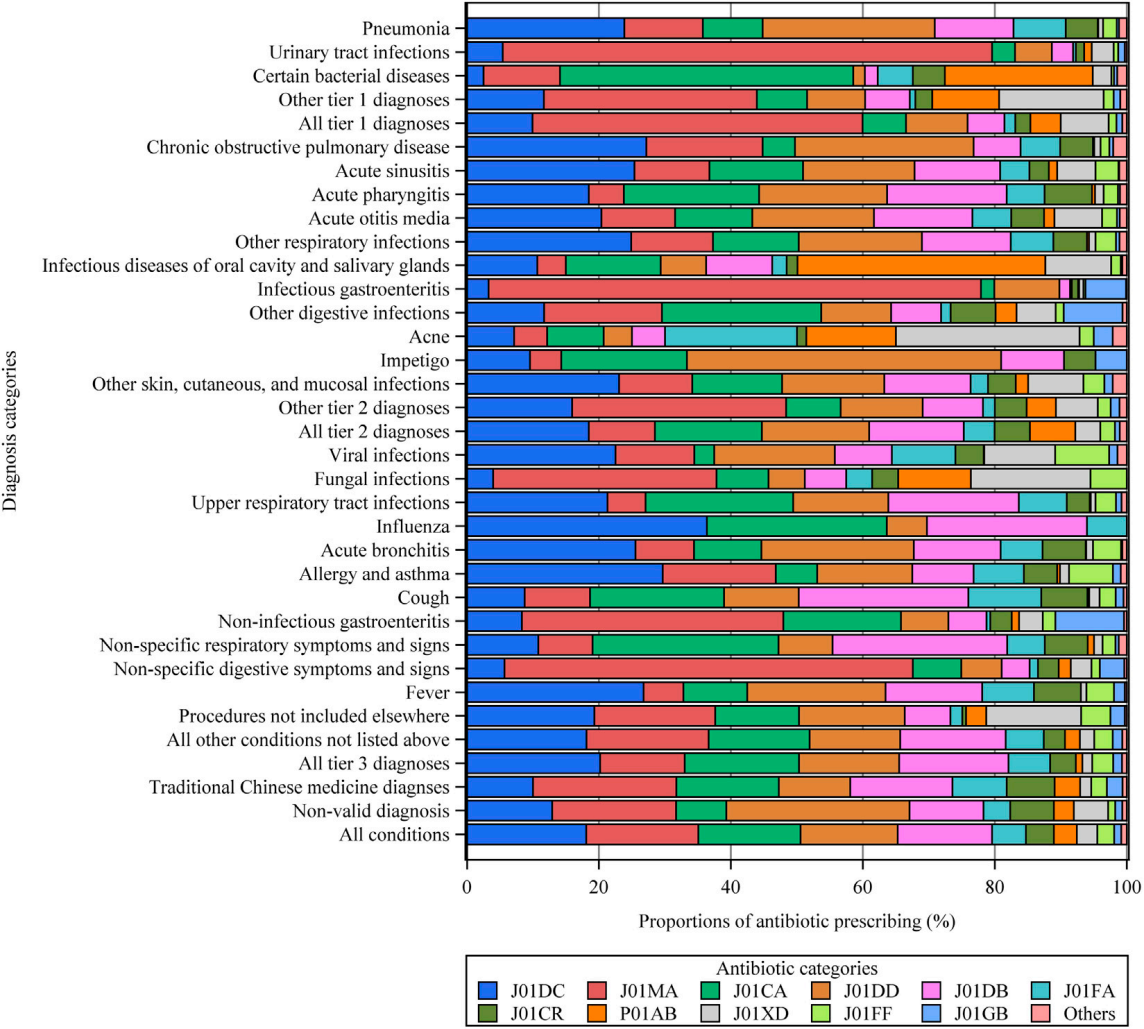
Types of antibiotics prescribed for different diagnostic categories. Other tier 1 diagnoses = other tier 1 bacterial infections. Other respiratory infections = other infectious diseases of the respiratory system categorized in tier 2. Other tier 2 diagnoses = other tier 2 infectious diseases for which an antibiotic may be indicated. See the Supplementary Table S2 for more details on all the diagnostic categories. J01CA = penicillins with extended spectrum. J01CR = combinations of penicillins, including beta-lactamase inhibitors. J01DB = first-generation cephalosporins. J01DC = second-generation cephalosporins. J01DD = third-generation cephalosporins. J01FA = macrolides. J01FF = lincosamides. J01MA = fluoroquinolones. J01GB = other aminoglycosides. J01XD = imidazole derivatives. P01AB = nitroimidazole derivatives. Others = include J01AA, J01CE, J01CF, J01EB, J01EE, and J01X.

A total of 1,416,120 individual antibiotics were prescribed, of which 1,087,630 (76.8%) were broad-spectrum antibiotics (Supplementary Table S7). Overall, Access antibiotics accounted for 45.0%, the Watch category accounted for 54.9%, while Reserve antibiotics were prescribed in only 16 visits (Figure 2). Among all diagnostic categories, the Watch group accounted for the highest proportion of antibiotics used to treat urinary tract infections (85.6%), infectious gastroenteritis (88.1%), and chronic obstructive pulmonary disease (77.8%). The most commonly prescribed antibiotics were second-generation cephalosporins (J01DC, 255,965, 18.1%), followed by fluoroquinolones (J01MA, 240,603, 17.0%), penicillins with extended spectrum (J01CA, 218,942, 15.5%), third-generation cephalosporins (J01DD, 208,695, 14.7%), and first-generation cephalosporins (J01DB, 202,607, 14.3%) (Figure 3). Fluoroquinolones accounted for the predominant proportion of all antibiotics in visits for several specific diagnoses, including urinary tract infections (74.2%), infectious gastroenteritis (74.6%), non-infectious gastroenteritis (39.6%), and non-specific symptoms and signs of the digestive system (61.9%). The most frequent individual antibiotics associated with appropriate, potentially appropriate, and inappropriate prescriptions are provided in the appendix (Supplementary Table S7).

### Impacts of the COVID-19 Pandemic

**FIGURE 4:**
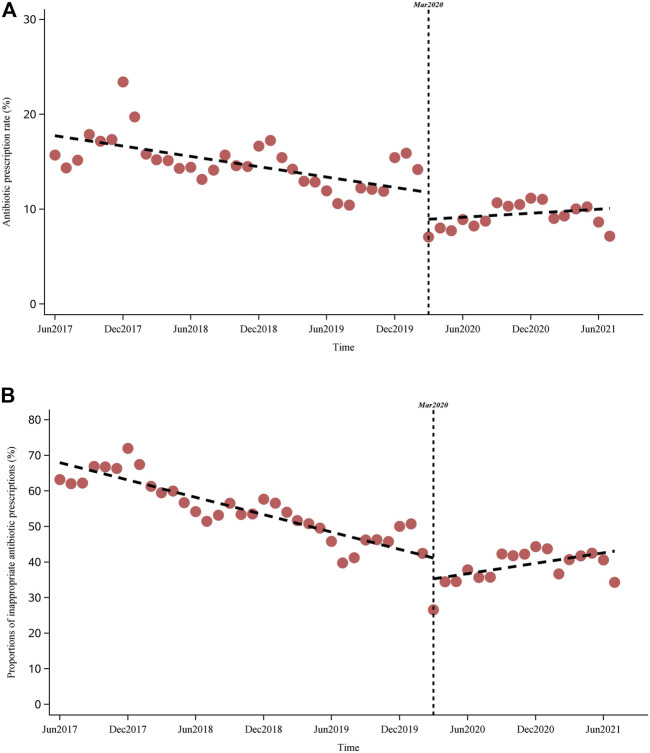
Impact of COVID-19 on the antibiotic prescription rate and inappropriate antibiotic prescriptions. (**A)** (above): Impact of COVID-19 on antibiotic prescription rate. (**B)** (below): Impact of COVID-19 on inappropriate antibiotic prescriptions.

A significant decreasing trend was observed in both the antibiotic prescription rates and proportions of inappropriate antibiotic prescriptions before the COVID-19 pandemic, with a monthly change of −0.2% (95% CI, −0.2 to −0.1) and −0.8% (−0.9 to −0.8), respectively (Figure 4 and Table 3). COVID-19 was associated with a 2.8% (−4.4 to −1.3) drop in level of and a 0.3% (0.2–0.3) increase in the monthly antibiotic prescription rate. Meanwhile, a −5.9% (−10.2 to −1.5) change in the level of and 1.3% (1.0–1.6) change in the monthly trend of inappropriate antibiotic prescriptions were associated with the COVID-10 pandemic. Further, a significant increasing trend was observed in inappropriate antibiotic prescriptions after March 2020 (slope 0.5% [0.2–0.8]). Similar results were observed in subgroups of different area types and institutional levels. For example, COVID-19 was associated with changes of −2.4% (−3.5 to −1.4) and −4.2% (−6.9 to −1.5) in the level of antibiotic prescription rates in urban and rural primary care institutions, as well as changes of −7.7% (−12.7 to −2.6) and −5.1% (−10.2 to −0.08) in the level of inappropriate antibiotic prescriptions in urban and rural primary care institutions, respectively (Table 3 and Supplementary Figures S1–S4). For different diagnostic categories, COVID-19 was associated with a level change of −2.2% (−4.2 to −0.2), −5.0% (−6.7 to −3.3), −2.1% (−3.6 to −0.6) and 0.2% (−0.3 to 0.7) in the antibiotic prescription rates for visits with tier 1, tier 2, tier 3, and TCM diagnoses, respectively (Supplementary Table S8).

**TABLE 3:** Trends of and impact of COVID-19 on the antibiotic prescriptions in primary care settings in Yinchuan City.

	Trend before COVID-19	Level Change	Slope Change	Trend after COVID-19
Antibiotic prescription rates				
Overall	−0.2 (−0.2 to −0.1)	−2.8 (−4.4 to −1.3)	0.3 (0.2–0.3)	0.07 (−0.03 to 0.2)
Area type				
Urban	−0.04 (−0.06 to −0.01)	−2.4 (−3.5 to −1.4)	0.1 (0.08–0.2)	0.1 (0.04–0.2)
Rural	−0.2 (−0.3 to −0.1)	−4.2 (−6.9 to −1.5)	0.5 (0.3–0.6)	0.2 (0.1–0.4)
Type of primary care				
CHSCs/THs	−0.3 (−0.3 to −0.2)	−3.4 (−4.9 to −2.0)	0.4 (0.3–0.5)	0.1 (0.04–0.2)
CHSSs/VCs	0.01 (−0.02 to 0.03)	−2.5 (−3.7 to −1.2)	0.08 (−0.02 to 0.2)	0.09 (−0.01 to 0.2)
Proportion of inappropriate antibiotic prescriptions				
Overall	−0.8 (−0.9 to −0.8)	−5.9 (−10.2 to −1.5)	1.3 (1.0–1.6)	0.5 (0.2–0.8)
Area type				
Urban	−0.5 (−0.6 to −0.4)	−7.7 (−12.7 to −2.6)	1.0 (0.7–1.4)	0.5 (0.1–0.9)
Rural	−0.9 (−1.0 to −0.9)	−5.1 (−10.2 to −0.08)	1.3 (1.0–1.7)	0.4 (0.02–0.8)
Type of primary care				
CHSCs/THs	−0.9 (−1.0 to −0.8)	−5.4 (−10.4 to −0.4)	1.3 (1.0–1.7)	0.4 (0.05–0.8)
CHSSs/VCs	−0.6 (−0.8 to −0.5)	−6.3 (−11.1 to −1.5)	1.1 (0.7–1.4)	0.4 (0.10–0.7)

CHSCs, City community health service centers; THs, Township hospitals; CHSSs, community health service stations; VCs, Village clinics.

## Discussion

Using a large prescription database with over 10 million outpatient visits, we measured antibiotic prescriptions in both urban and rural primary care facilities in a Chinese city. We estimated that 12.6% of outpatient visits in primary care resulted in antibiotic prescriptions during the study period, whereas 50.7% of these antibiotic prescriptions were considered inappropriate. This study extended the evidence from our previous studies on inappropriate antibiotic prescriptions in Chinese secondary and tertiary hospitals (Zhao et al., 2021a; Zhao et al., 2021b) and added valuable data on the appropriateness of antibiotic prescriptions in LMICs (Sulis et al., 2020a). Furthermore, to the best of our knowledge, this study was the first to assess the impact of the COVID-19 pandemic on antibiotic prescriptions in primary care settings in China. Our results indicated that the COVID-19 pandemic was associated with decreasing changes in antibiotic prescription rates and inappropriate antibiotic prescriptions.

A survey conducted 10 years ago estimated that 52.9% of outpatient visits in CHSCs and THs in six provinces of China received antibiotics, and 60.4% of these antibiotic prescriptions were inappropriate (Wang et al., 2014). Compared with these results, our findings that 12.6% of outpatient visits resulted in antibiotic prescriptions and 50.7% of antibiotics were prescribed inappropriately during the study period indicated that antibiotic use in Chinese primary care settings might have considerably improved in recent years, which was reflected by the decreasing trends in the antibiotic prescription rates and proportions of inappropriate antibiotic prescriptions before March 2020 in this study. A series of interventions and policies have been introduced to restrain the overuse and misuse of antibiotics in China in the past decade (Zhao et al., 2021a). Several studies have demonstrated that these policies, especially the long-term national antimicrobial stewardship campaign started in 2011, which stipulated that the percentage of outpatient prescriptions containing antibiotics should not exceed 20%, have dramatically reduced antibiotic prescription rates in primary care institutions (Lin et al., 2016; Li et al., 2020; Gong et al., 2021). Although indirectly, this study added valuable knowledge on the changes in inappropriate antibiotic prescriptions after the antimicrobial stewardship in Chinese primary care settings. However, evidence on the impact of these policies on patient care outcomes or patient satisfaction is scarce. Two other studies, including multiple primary care institutions, to assess the appropriateness of antibiotic prescriptions have been performed in recent years. Among these studies, one showed that 84.1% of antibiotic prescriptions in THs in Guizhou province in 2018 were considered unnecessary (Chang et al., 2019), while another study found that 12.5% of antibiotic prescriptions in CHSCs and CHSSs in the Beijing Dongcheng District between 2015 and 2018 were rated as inappropriate (Taxifulati et al., 2021). Both of these studies were conducted through manual prescription reviews, which have not been clearly described and validated. Furthermore, none of the studies covered all four types of primary care institutions, posing a high risk of biased estimation of the proportion of inappropriate antibiotic prescriptions, as available evidence demonstrates that primary care in rural areas and lower-level primary care institutions tend to prescribe antibiotics more inappropriately (Yau et al., 2021).

The increasing use of antibiotics in LMICs has been the most important driver of the dramatically increasing global consumption of antibiotics (Klein et al., 2018), with the majority occurring in primary care settings in many countries (Dyar et al., 2016; Peñalva et al., 2020). However, the appropriateness and pattern of antibiotic use in outpatient primary care settings has not been well assessed in LMICs (Sulis et al., 2020a). This information is crucial in antimicrobial stewardship for designing and implementing interventions and policies (Pulcini and Hulscher, 2019; Sulis et al., 2020a). A recent systematic review showed that as many as 52% of outpatients in primary care visits receive antibiotic prescriptions in 27 LMICs; however, only nine studies assessed the rationality of antibiotic prescriptions, with highly variable estimates of the proportion of inappropriate prescriptions, ranging from 8 to 100% (Sulis et al., 2020a). Furthermore, most of these studies focused only on specific conditions, such as acute respiratory tract infection and diarrhea, and had small sample sizes and methodological issues (Sulis et al., 2020a). Studies using standardized patients who presented with specific diseases that never indicated antibiotics showed that 42–50% of antibiotics were inappropriately prescribed across primary care settings in China, India, and Kenya (Xue et al., 2019; Sulis et al., 2020b), similar to our results. However, the over- and mis-prescription of antibiotics is not confined to LMICs. Two studies in the United States using the Veterans Affairs Healthcare System reported that 50% of antibiotic prescriptions in primary care clinics were considered unnecessary (Shively et al., 2018; Kiel et al., 2020). In Canada, the antibiotic prescription rate for 23 specific conditions is 30.6 and 24.3% of these antibiotics are prescribed for conditions that never or rarely justify the use of antibiotics (Schwartz et al., 2020). However, up to 23% of antibiotic prescriptions in United Kingdom primary care settings are considered inappropriate (Smieszek et al., 2018). Owing to the great differences in methodology and data, direct comparisons among studies in different countries and regions are not feasible.

Similar to our previous findings in secondary and tertiary hospitals (Zhao et al., 2021a), tier 3 respiratory diseases were important drivers of inappropriate antibiotic prescriptions in primary care. Among these conditions, acute bronchitis and upper respiratory tract infections ranked in the top two with the highest antibiotic prescription rates. However, a comparably high level of antibiotic prescriptions was found in some developed countries, where 82% of visits with acute bronchitis, 25% of visits for upper respiratory tract infections in the United Kingdom, and 61% of visits for acute bronchitis in Canada received antibiotic prescriptions in primary care facilities (Pouwels et al., 2018; Schwartz et al., 2020). Although defined as potentially appropriate, a substantial proportion of antibiotics for tier 2 conditions may be unnecessary. For example, 88, 68, and 62% of outpatient visits with diagnoses of acute otitis media, acute sinusitis, and acute pharyngitis, respectively, resulted in antibiotic prescriptions in primary care, whereas the ideal prescription rates for these conditions are less than 20% (Adriaenssens et al., 2011; Pouwels et al., 2018). In contrast, tier 1 conditions that almost always warrant antibiotics were undertreated in primary care, with only 57.5% of visits for pneumonia and 40.3% of visits for diagnoses of bacterial infections resulting in antibiotic prescriptions. However, similar results were found in Chinese secondary healthcare settings (Zhao et al., 2021a), as well as in primary care settings of some developed countries (Schwartz et al., 2020). In addition, antibiotic selection appeared to lack appropriateness in primary care facilities, with the Watch group, which is only indicated for a specific, limited number of infective syndromes, and is more prone to be a target of antibiotic resistance, accounted for 55% of all antibiotics. In contrast, the proportion of antibiotics in the Watch category was <40% in some LMICs (Sulis et al., 2020a). However, a rapid increase in Watch antibiotic consumption has been observed in many countries, particularly in LMICs, reflecting the priority of antibiotic stewardship targeting Watch antibiotics (Klein et al., 2021). Furthermore, nearly 80% of prescribed antibiotics belong to broad spectrum in the primary care setting, which was also observed in Chinese secondary and tertiary hospitals (Zhao et al., 2021a). For example, fluoroquinolones accounted for 74.2% of antibiotics used for urinary tract infections, which is recommended to be lower than 5% in European countries (Adriaenssens et al., 2011). Similar prescription patterns have been observed for primary care facilities in other regions of China (Gong et al., 2021; Taxifulati et al., 2021). The overuse of broad-spectrum antibiotics can have a crucial impact on AMR, indicating the need to improve the rationality of antibiotic types in China’s future antibiotic stewardship campaign (Gong et al., 2021).

We found that some institutional, patient, and physician characteristics might be associated with inappropriate antibiotic dispensing. Primary care institutions in urban areas and higher-level institutions of CHSCs/THs were associated with fewer inappropriate antibiotic prescriptions, probably because they were better equipped and had more experienced healthcare professionals (Li et al., 2017; National Health Commission of the People’s Republic of China, 2018), which is important for accurate diagnosis and appropriate prescription (Xue et al., 2019). Previous studies have also demonstrated that rurality is an independent risk factor for inappropriate prescriptions (Sulis et al., 2020b; Yau et al., 2021). We also found that children under 18 years of age and male patients were more likely to receive inappropriate antibiotic prescriptions, which is different from the practice in some developed countries where younger age and female gender of patients are associated with inappropriate prescribing practices (Singer et al., 2018; Yau et al., 2021). The improved health-seeking behavior of parents for their children and the prevalent use of antibiotics by physicians for fear of adverse consequences of delayed treatment might be important factors influencing high antibiotic use and inappropriate antibiotic prescriptions among children (Fink et al., 2020). In addition, our results indicated that female physicians aged >60 years prescribed fewer inappropriate antibiotics, while a higher education level was associated with a slightly higher rate of inappropriate antibiotic prescriptions after adjusting for other factors. Although physician knowledge and behavior can have crucial impacts on antibiotic use, other internal and external determinants, including apprehension of complications because of undertreatment, patients’ expectations for antibiotics, and intentions to maintain good relations, as well as financial incentives from drug sales, have also been proven to induce physicians to prescribe antibiotics inappropriately (Yau et al., 2021). Our findings may have suffered from some residual confounding and suggest that further studies are needed to clarify the underlying factors influencing these associations. However, this study provides potential targets for future antibiotic stewardship to reduce inappropriate antibiotic use in primary care settings in China. Systematic antibiotic stewardship strategies in primary care settings that focus on physician and patient education and clinician support systems, as well as monitoring of antibiotic prescriptions and AMR and policy changes, are needed in LMICs, including China (Hawes et al., 2020; Yau et al., 2021).

We found that the COVID-19 pandemic was associated with a decrease in overall and inappropriate antibiotic prescriptions in Chinese primary care facilities, with immediate levels decreasing in March 2020, and increasing trends thereafter. Similar effects have been observed in other studies (Buehrle et al., 2020; King et al., 2020; Lepak et al., 2021; Miller et al., 2021). The COVID-19 pandemic has brought about a wide range of changes in the structure of health services, health-seeking behaviors, and medication supplies (Knight et al., 2021). Measures implemented to prevent and control the spread of COVID-19, including social isolation, use of personal protective equipment, hand and environmental hygiene, and active identification and quarantine of close contacts, have been effective in reducing the incidence of other infectious diseases, thus indirectly reducing antibiotic prescriptions for both necessary and unnecessary use (Buehrle et al., 2020; King et al., 2020; Parry et al., 2020; Knight et al., 2021; Lai et al., 2021; Lepak et al., 2021; Miller et al., 2021). Early in the pandemic, antibiotics and other anti-inflammatory agents were partially unavailable in pharmacies and primary care institutions in China, and physicians prescribed these drugs carefully, fearing delays in identifying potential COVID-19 patients because of patient self-medication or insufficient diagnostic capabilities in primary care facilities. This might have led to delayed seeking of treatment or referrals to secondary and tertiary hospitals in patients with infectious diseases, resulting in a decrease in the presentation of infectious diseases and the need for antibiotic prescriptions in primary care settings. The increasing trend of inappropriate antibiotic prescriptions after the effective control of COVID-19 was potentially associated with the gradual return of medical order. However, further studies are required to explain this change. Furthermore, numerous studies have shown that a high proportion of COVID-19 patients are treated with antibiotics during hospitalization (Knight et al., 2021; Lai et al., 2021), indicating that the COVID-19 pandemic might have different effects on antibiotic use and AMR in secondary and tertiary hospitals where COVID-19 patients are mainly admitted in China. Thus, as the COVID-19 pandemic progresses, more detailed and extensive studies of antibiotic use and AMR in China, LMICs, and globally are urgently needed.

This study has several strengths. We provided the most recent and comprehensive data on antibiotic prescriptions in primary care settings in a mainland China city using an unprecedentedly large sample of prescription data. The method for assessing the appropriateness of antibiotic prescriptions used in this study was mainly based on the ICD-10, suffering little effect of personal knowledge and experience. This method has been well validated (Fleming-Dutra et al., 2016; Chua et al., 2019; Zhao et al., 2020) and can be used in other healthcare settings in LIMCs. With the improvement in the digitization of medical information and quality of diagnostic coding in primary care institutions, this method could be integrated into an automatic monitoring system to regularly evaluate the appropriateness of antibiotic prescriptions. In addition, we applied interrupted time series analysis, which is considered the strongest quasi-experimental research design (Penfold and Zhang, 2013), to evaluate the impact of COVID-19 on antibiotic prescriptions in primary care settings, providing more evidence of the broad impacts of COVID-19. However, this study has some limitations. First, prescription data were only from primary care institutions in a single city, and thus may not reflect antibiotic use in the primary care of the whole country. As large regional variations presented in antibiotic use (Li et al., 2020), additional studies using more representative data are needed. Second, the appropriateness of antibiotic use is a composite measure based on multiple factors including indications, antibiotic choice, route of administration, and treatment duration. Nevertheless, as in previous studies (Fleming-Dutra et al., 2016; Chua et al., 2019; Zhao et al., 2021a), only indications were considered in the present study. This might have led to the underestimation of inappropriate prescriptions. Third, a limited number of potential influencing factors at the institutional, patient, and physician levels were collected in the database; thus, caution should be exercised when interpreting our results. Additional potential determinants of inappropriate antibiotic prescriptions, such as patient education level and knowledge of antibiotics, communication and interaction between physicians and patients, and staffing and equipment of medical institutions, need to be further studied in future studies.

In conclusion, the antibiotic prescription rate was under the required control level, and inappropriate antibiotic prescription tended to decrease gradually in primary care settings in the city of Yinchuan. However, inappropriate antibiotic prescriptions are still highly prevalent in Chinese primary care settings, with over half of all antibiotics prescribed inappropriately during the study period. A systematic antibiotic stewardship program focusing on multiple factors is needed to further optimize antibiotic prescriptions in primary care settings. Furthermore, the COVID-19 pandemic might have had an effect on reducing antibiotic prescription rates and inappropriate antibiotic prescriptions in primary care settings.

## Data Availability

Data used in this study are available to the scientific community and the requests should be sent to the corresponding authors.
