# Supplementary material for: Appropriateness of Antibiotic Prescriptions in Chinese Primary Health Care and the Impact of the COVID-19 Pandemic: A Typically Descriptive and Longitudinal Database Study in Yinchuan City
Source: Front Pharmacol. 2022 Apr 14;13:861782. doi: 10.3389/fphar.2022.861782 (PMC9049214; doi:10.3389/fphar.2022.861782)
Supplement: Supplementary file 1 [file DataSheet1.pdf]

Appendix Table of Contents

Table S1. Antibiotics assessed in this study..2
Table S2. Categories of diagnoses within different tiers.. 3
Table S3. Antibiotic prescription rates for various diagnosis categories in Chines primary care setting..10
Table S4. Antibiotic prescription rates for various subgroups of patient visits in Chines primary care setting...12
Table S5. Sensitivity analyses for proportion of antibiotic prescriptions in each appropriateness category, all TCM diagnoses classified as tier 2 or tier 3..14
Table S6. Standardized proportion of antibiotic prescriptions in each appropriateness category for various subgroups...15
Table S7. Most commonly prescribed antibiotics for different diagnosis categories.......................17
Figure S1. Impact of COVID-19 on the antibiotic prescription rate in the urban and rural primary care setting...18
Figure S2. Impact of COVID-19 on the antibiotic prescription rate in the CHSCs/THs and CHSSs/VCs.. 18
Figure S3. Impact of COVID-19 on the proportion of inappropriate antibiotic prescriptions in the urban and rural primary care setting...19
Figure S4. Impact of COVID-19 on the proportion of inappropriate antibiotic prescriptions in the CHSCs/THs and CHSSs/VCs..19
Table S8. Impact of COVID-19 on the antibiotic prescription rates in visits for different diagnosis categories...20
References.. 21

Table S1. Antibiotics assessed in this study

Antibiotic categories	Antibiotics[a,b]
**Parenteral antibiotics**[c]	
Beta-lactam antibacterials, penicillins	Amoxicillin-clavulanate, Azlocillin , Benzathine benzylpenicillin, Benzylpenicillin, Mezlocillin, Piperacillin-sulbactam
Other beta-lactam antibacterials	Cefathiamidine, Cefazolin, Cefmetazole, Cefminox, Cefoperazone-sulbactam, Cefoperazone-tazobactam, Cefotaxime, Cefotiam, Cefoxitin, Cefradine, Ceftazidime, Ceftezole, Ceftizoxime, Ceftriaxone, Cefuroxime
Macrolides and lincosamides	Azithromycin, Clindamycin , Erythromycin
Aminoglycoside Antibacterials	Amikacin, Etimicin, Gentamicin
Quinolone antibacterials	Ciprofloxacin, Levofloxacin, Moxifloxacin, Pazufloxacin
Other antibacterials	Fosfomycin, Metronidazole, Ornidazole
**Oral antibiotics**	
Tetracyclines	Doxycycline, Tetracycline
Beta-lactam antibacterials, penicillins	Amoxicillin, Amoxicillin-clavulanate, Ampicillin, Oxacillin
Other beta-lactam antibacterials	Cefaclor, Cefadroxil, Cefalexin, Cefdinir, Cefetamet, Cefixime, Cefprozil, Cefradine, Cefuroxime
Sulfonamides and trimethoprim	Sulfamethoxazole-trimethoprim, Sulfapyridine
Macrolides and lincosamides	Azithromycin, Clarithromycin, Clindamycin , Dirithromycin , Erythromycin, Erythromycin cyclocarbonate, Erythromycin estolate, Erythromycin ethylsuccinate, Roxithromycin
Aminoglycoside Antibacterials	Gentamicin
Quinolone antibacterials	Ciprofloxacin, Levofloxacin, Moxifloxacin, Norfloxacin
Nitroimidazole derivatives	Metronidazole, Ornidazole, Tinidazole

a Antibiotics were coded in accordance with the Anatomical Therapeutic Chemical (ATC) classification system.

b Except metronidazole, ornidazole, tinidazole, and furazolidone, all other antibiotics are classified as ‘J01’.

c Parenteral antibiotics included intravascular and intramuscular antibiotics.

Table S2. Categories of diagnoses within different tiers

Details of establishing these diagnosis categories can be found in our previous studies.[1, 2]

Categories of diagnosis	ICD-10 codes and description
**Tier 1 diagnoses that antibiotics are almost always indicated**	
Certain bacterial diseases	A01 Typhoid and paratyphoid fevers; A02.1 Salmonella sepsis; A04.7 Enterocolitis due to Clostridium difficile; A06 Amebiasis; A15–A19 Tuberculosis; A20–A28 Certain zoonotic bacterial diseases; A30–A49 Other bacterial diseases (except A49.800–A49.801, A49.900); A50 Congenital syphilis; A51 Early syphilis; A52 Late syphilis; A53 Other and unspecified syphilis; A54 Gonococcal infection; A55 Chlamydial lymphogranuloma (venereum); A56 Other sexually transmitted chlamydial diseases; A57 Chancroid; A58 Granuloma inguinale; A59 Trichomoniasis; A65–A69 Other spirochaetal diseases; A70–A74 Other diseases caused by chlamydiae; A75–A79 Rickettsioses; B58 Toxoplasmosis; B59 Pneumocystosis; B60 Other protozoal diseases, not elsewhere classified (except B60.8); B90 Sequelae of tuberculosis (except B90.001, B90.101, B90.201, B90.801–B90.803, B90.901–B90.904); B92 Sequelae of leprosy; B94.0 Sequelae of trachoma; B95 Streptococcus, Staphylococcus, and Enterococcus as the cause of diseases classified elsewhere; B96 Other bacterial agents as the cause of diseases classified elsewhere (except B96.2); Sepsis classified elsewhere (e.g., P36 Bacterial sepsis of newborn); Other specific bacterial infectious disease (e.g., P37.0 Congenital tuberculosis); R65.201 Infectious shock; U80, U81, U88, U89 Drug-resistance bacteria.
Pneumonia	J13–J18 Pneumonia (except viral and fungal pneumonia); Pneumonia due to specific bacterial and classified elsewhere (e.g., P23.1–P23.6 Congenital pneumonia due to specific bacterial).
Urinary tract infections	N10.x02 Acute pyelonephritis; N13.6 Pyonephrosis; N15.1 Renal and perinephric abscess; N15.901 Kidney infection; N28.834 Ureteritis; N30.0 Acute cystitis; N30.3 Trigonitis; N30.801 Abscess of bladder; N34.0 Urethral abscess; N34.101 Non-gonorrheal urethritis; N34.204, N34.205 Acute and chronic urethritis; N39.0 Urinary tract infection, site not specified (except N39.001).
Other bacterial infections	D73.0 Abscess of spleen; E06.002 Abscess of thyroid; E10.505, E11.505 Diabetic peripheral angiopathy with gangrene; E23.601 Abscess of pituitary gland; E27.802 Abscess of adrenal gland; E32.1 Abscess of thymus; G00 Bacterial meningitis, not elsewhere classified; G01 Meningitis in bacterial diseases classified elsewhere; G04.2 Bacterial meningoencephalitis and meningomyelitis, not elsewhere classified; G04.805 Suppurative encephalitis; G04.807 Suppurative meningitis; G04.901 Suppurative ventriculitis; G06 Intracranial and intraspinal abscess and granuloma; G07 Intracranial and intraspinal abscess and granuloma in diseases classified elsewhere; H05.0 Acute inflammation of orbit (except H05.002, H05.005 classified as tier 2); H60.2 Malignant otitis externa; H70, H75.0, H95.1 Mastoiditis and related conditions (except H70.004); I00 Rheumatic fever without heart involvement; I01 Rheumatic fever with heart involvement; I02 Rheumatic chorea; I30.101–I30.102 Suppurative and bacterial

	pericarditis; I33 Acute and subacute endocarditis (except I33.008–I33.102 were classified as tier 2); I40.002 Acute bacterial myocarditis; I70.202, I70.205, I70.207 Atherosclerosis of arteries with gangrene; I77.806 Abscess of aorta; I96 Gangrene, not elsewhere classified; R02.x00 Gangrene; R65.201 Infectious shock; J32.004, J32.102, J32.802, J32.905 Abscess of sinuses; J34.007 Abscess of nasal septum; J36 Peritonsillar abscess; J38.305, J38.707, J38.716 Abscess of vocal cords, larynx, and epiglottis; J39.0 Retropharyngeal and parapharyngeal abscess; J39.1 Other abscess of pharynx; J85 Abscess of lung and mediastinum; J86 Pyothorax; J98.501, J98.503 Mediastinitis; K04.003 Abscess of pulp; K04.6 Periapical abscess with sinus; K04.7 Periapical abscess without sinus; K05.201–K05.203 Periodontal abscess; K11.3 Abscess of salivary gland; K12.2 Cellulitis and abscess of mouth; K14.001 Abscess of tongue; K22.3 Perforation of esophagus; K25.1–K25.2, K25.5–K25.6, K26.1–K26.2, K26.5–K26.6, K27.1–K27.2, K27.5–K27.6, K28.1–K28.2, K28.5–K28.6 Peptic ulcer with perforation; K35–K37 Appendicitis; K40.1, K40.4, K41.1, K41.4, K42.1, K43.1, K44.1, K45.1, K46.1 Abdominal hernia, with gangrene (all other hernias were classified as tier 3); K55.005 Mesenteric gangrene; K57.0 Diverticulitis of small intestine with perforation and abscess; K57.106–K57.108 Diverticulitis of small intestine and duodenum; K57.2 Diverticulitis of large intestine with perforation and abscess; K57.304–K57.305 Diverticulitis of colon and cecum; K57.4 Diverticulitis of both small and large intestine with perforation and abscess; K57.8 Diverticulitis of intestine, part unspecified, with perforation and abscess; K63.0 Abscess of intestine; K63.1 Perforation of intestine; K65 Peritonitis (except K65.804, K65.806); K75.0 Abscess of liver; K80.0–K80.4 Cholelithiasis with cholecystitis; K81 Cholecystitis; K82.2 Perforation of gallbladder; K83.0 Cholangitis; K83.2 Perforation of bile duct; K85.817–K85.818 Acute suppurative pancreatitis; K85.824–K85.825Abscess of pancreas; K90.1 Tropical sprue. L00 Staphylococcal scalded skin syndrome; M00 Pyogenic arthritis; M46.2 Osteomyelitis of vertebra; M46.3 Infection of intervertebral disc (pyogenic); M46.501–M46.504, M46.801 Abscess of spinal column; M60.005, M60.006, M60.008 Abscess of muscle; M65.0 Abscess of tendon sheath; M65.1 Other infective (teno) synovitis; M72.6 Necrotizing fasciitis; M86 Osteomyelitis (except M86.801, M86.804, M86.805, M86.809, M86.921, M86.923); M94.805 Abscess of cartilage; N41.0 Acute prostatitis; N41.2 Abscess of prostate; N43.1 Infected hydrocele; N45.0 Orchitis and epididymitis with abscess; N48.201, N48.203 Cellulitis abscess of penis; N49.102 Abscess of tunica vaginalis; N49.201–N49.202 Cellulitis and gangrene of scrotum; N49.204 Abscess of scrotum; N70 Salpingitis and oophoritis (except N70.103, B70.104); N71 Inflammatory disease of uterus, except cervix; N72 Inflammatory disease of cervix uteri; N73 Other female pelvic inflammatory diseases (except N73.6, N73.801); N74 Female pelvic inflammatory disorders in diseases classified elsewhere; N98.0 Infection associated with artificial insemination; O03.0, O03.5, O04.0, O04.5, O05.0, O05.5, O06.0, O06.5, O07.0, O07.5, O08.0 Genital tract and pelvic infection following ectopic, molar pregnancy or termination of pregnancy;

	O04.804 Septic shock following termination of pregnancy; O23 Infections of genitourinary tract in pregnancy (except O23.503, O23.503, O23.505, O23.506); O41.1 Infection of amniotic sac and membranes; O85 Puerperal sepsis; O86 Other puerperal infections (except O86.4); O99.6 Diseases of the digestive system complicating pregnancy, childbirth and the puerperium; P02.7 Newborn affected by chorioamnionitis; P38 Omphalitis of newborn; P39 Other infections specific to the perinatal period (except P39.402, P39.403, P39.8, P39.9); P77 Necrotizing enterocolitis of newborn; Pyemic and septic embolism classified elsewhere (e.g., O88.3 Obstetric pyemic and septic embolism); Open fracture (e.g., S02.011 Open fracture of frontal bone; S02.012 Open fracture of Parietal bone, etc.); Open injury of visceral organs (e.g., S06.211 Open injury of brain, S06.410 Open epidural hemorrhage; S36.310 Open rupture of stomach, etc.); S05.7 Avulsion of eye; Avulsion and traumatic amputation of part of body (e.g., S08 Avulsion and traumatic amputation of part of head, etc.); T80.2 Infections following infusion, transfusion and therapeutic injection; Infection and inflammatory reaction due to prosthetic devices, implants and grafts (e.g., T82.7 Infection and inflammatory reaction due to other cardiac and vascular devices, implants and grafts); T87.4 Infection of amputation stump; T87.Necrosis of amputation stump; Bitten by animals (e.g., W53.x00 Bitten by rat, etc.).
**Tier 2 diagnoses that antibiotics are sometimes indicated**	
Acute otitis media	H66 Suppurative and unspecified otitis media.
Acute pharyngitis	J02 Acute pharyngitis (except J02.0 was classified as tier 1); J03 Acute tonsillitis (except J03.0 was classified as tier 1).
COPD	J44 Other chronic obstructive pulmonary disease.
Acute sinusitis	J01 Acute sinusitis.
Other infectious diseases of the respiratory system	J04.001 Acute suppurative laryngitis; J04.1 Acute tracheitis; J05.1 Acute epiglottitis; J10.000, J10.100, J11.000, J11.100 Influenza due to other identified influenza virus with pneumonia; J22 Unspecified acute lower respiratory infection; J31.2 Chronic pharyngitis; J32 Chronic sinusitis; J34.0 Abscess, furuncle and carbuncle of nose (include J34.806); J35.0 Chronic tonsillitis and adenoiditis; J41 Simple and mucopurulent chronic bronchitis; J42 Unspecified chronic bronchitis; J43 Emphysema; J47 Bronchiectasis; J69.001 Inhaled pneumonia; J90 Pleural effusion, not elsewhere classified; J91 Pleural effusion in conditions classified elsewhere; J95 Intraoperative and postprocedural complications and disorders of respiratory system, not elsewhere classified; J98 Other infectious diseases of lung, thoracic cavity, and mediastinum (except J98.0–J98.3, J98.6, J98.9).
Infectious diseases of oral cavity and salivary glands	K04.0 Pulpitis; K04.1 Necrosis of pulp; K04.2 Pulp degeneration; K04.3 Abnormal hard tissue formation in pulp; K04.4 Acute apical periodontitis of pulpal origin; K04.5 Chronic apical periodontitis; K05 Gingivitis and periodontal diseases; K10.3 Alveolitis; K11.2 Sialoadenitis; K12.111 Oral infection; K13.011 Cellulitis of lips.
Infectious gastroenteritis	A00 Cholera; A02.0 Salmonella enteritis; A03 Shigellosis; A04 Other bacterial

	intestinal infections (except A04.3, A04.7); A05 Other bacterial foodborne intoxications; A07 Other protozoal intestinal diseases; A09 Infectious gastroenteritis and colitis, unspecified.
Other infectious diseases of the digestive system	K20 Esophagitis; K25 Gastric ulcer (except conditions of perforation); K26 Duodenal ulcer (except conditions of perforation); K27 Peptic ulcer, site unspecified (except conditions of perforation); K31.6 Fistula of stomach and duodenum; K38.3 Fistula of appendix; K50 Crohn's disease; K51 Ulcerative colitis; K55 Vascular disorders of intestine (conditions with gangrene were classified as tier 1 and except K55.1, K55.2, K55.8, K55.9); K60 Fissure and fistula of anal and rectal regions; K61 Abscess of anal and rectal regions; K63.2 Fistula of intestine; K75.1 Phlebitis of portal vein; K82.0 Obstruction of gallbladder; K82.3 Fistula of gallbladder; K83.1 Obstruction of bile duct; K83.3 Fistula of bile duct; K91.8 Other intraoperative and postprocedural complications and disorders of digestive system (except K91.800–K91.801, and K91.803–K91.806).
Impetigo	L01 Impetigo.
Acne	L70 Acne.
Other infectious diseases of the skin and subcutaneous tissue	L02 Cutaneous abscess, furuncle and carbuncle; L03 Cellulitis and acute lymphangitis; L04 Acute lymphadenitis; L05 Pilonidal cyst and sinus; L08 Other local infections of skin and subcutaneous tissue; L30.3 Infective dermatitis; L60.0 Ingrowing nail; L71 Rosacea; L73.804, L73.805 Ficosis; L88 Pyoderma gangrenosum; L89 Pressure ulcer; L97 Non-pressure chronic ulcer of lower limb, not elsewhere classified; L98 Other disorders of skin and subcutaneous tissue, not elsewhere classified.
Other infectious diseases that antibiotic may be indicated	A02.2 Localized salmonella infections; A02.8–A02.9 Other specified or unspecified salmonella infections; A49.800–A49.801, A49.900 Other bacterial infection, unspecified; A64 Unspecified sexually transmitted disease; B55 Leishmaniasis (except B55.0); B60.8 Other specified protozoal diseases; B64 Unspecified protozoal disease; B85 Pediculosis and phthiriasis; B89 Unspecified parasitic disease; B99 Other and unspecified infectious diseases; Bloodstream infections; Infectious diseases that bacterial not specified; Suppurative diseases not classified elsewhere; D57.0 Sickle-cell disorders with crisis; D57.8 Other sickle-cell disorders; D70 Neutropenia; E06.0, E06.9 Acute thyroiditis (except E06.002); E84.0 Cystic fibrosis with pulmonary manifestations; E84.9 Cystic fibrosis, unspecified; G03.9 Meningitis, unspecified (except G03.907); G04 Encephalitis, myelitis and encephalomyelitis (except G04.2 and G04.805–G04.807); G05 Encephalitis, myelitis and encephalomyelitis in diseases classified elsewhere; G06.006 Intracranial infection; G08 Intracranial and intraspinal phlebitis and thrombophlebitis; H00.2–H00.3 Abscess and cellulitis of eyelid; H01.0 Blepharitis; H02.808 Retained foreign body in eyelid; H04.0 Dacryoadenitis; H04.3 Acute and unspecified inflammation of lacrimal passages; H04.4 Chronic inflammation of lacrimal passages; H05.1 Chronic inflammatory disorders of orbit; H05.5 Retained (old) foreign body following penetrating wound of orbit; H16 Keratitis; H20 Iridocyclitis; H30 Chorioretinal inflammation; H44.0 Purulent

	endophthalmitis; H44.1 Other endophthalmitis; H44.6–H44.7 Retained (old) intraocular foreign body; H46 Optic neuritis; H60 Otitis externa (except H60.2); H61.0 Chondritis and perichondritis of external ear; H72 Perforation of tympanic membrane; H73 Other disorders of tympanic membrane (except H73.800, H73.801, H73.804, H73.9); H83.0 Labyrinthitis; I30 Acute pericarditis; I32 Pericarditis in diseases classified elsewhere; I39 Endocarditis and heart valve disorders in diseases classified elsewhere; I40 Acute myocarditis; I41.0 Bacterial myocarditis in diseases classified elsewhere; I80 Phlebitis and thrombophlebitis; I88 Nonspecific lymphadenitis (except I88.0); I89.1 Lymphangitis; M02 Postinfective and reactive arthropathies; M46.4 Discitis, unspecified; M46.500 Other infective spondylopathies; M54.0 Panniculitis affecting regions of neck and back; M60.0 Myositis (except M60.005, M60.006, M60.008); M60.8 Other myositis; M65.0 Abscess of tendon sheath; M60.9 Myositis, unspecified; M71.0 Abscess of bursa; M71.1 Other infective bursitis; M79.3 Panniculitis, unspecified; M79.5 Residual foreign body in soft tissue; M94.801 Suppurative chondritis; N10.x00 Acute tubulo-interstitial nephritis; N11 Chronic tubulo-interstitial nephritis; N12.x02 Pyelonephritis; N13.204 Hydronephrosis with calculous pyelonephritis; N13.7 Vesicoureteral-reflux; N20.901 Pyelonephritis due to urinary calculus; N28.8 Other specified disorders of kidney and ureter; N30.1 Interstitial cystitis (chronic); N30.2 Other chronic cystitis; N30.8 Other cystitis (except N30.801); N30.9 Cystitis, unspecified; N32.1 Vesicointestinal fistula; N34.1 Nonspecific urethritis; N34.2 Other urethritis (except N34.204, N34.205); N39.001 Asymptomatic bacteriuria; N41.1 Chronic prostatitis; N41.3 Prostatocystitis; N41.8 Other inflammatory diseases of prostate; N41.9 Inflammatory disease of prostate, unspecified; N45.9 Orchitis and epididymitis; N48.1 Balanitis; N48.2 Other inflammatory disorders of penis; N48.5 Ulcer of penis; N49 Inflammatory disorders of male genital organs, not elsewhere classified (except N49.102, N49.201, N49.202, and N49.204); N49.9 Inflammatory disorder of unspecified male genital organ; N61 Inflammatory disorders of breast (including the N61.x00，N61.x04–N61.x05); N75.0 Cyst of Bartholin's gland; N75.1 Abscess of Bartholin's gland; N75.802 Inflammation of Bartholin's gland; N75.9 Disease of Bartholin's gland, unspecified; N76 Other inflammation of vagina and vulva; N77 Vulvovaginal ulceration and inflammation in diseases classified elsewhere; N82 Fistulae involving female genital tract; N85.8 Other specified noninflammatory disorders of uterus; O08.3 Shock following ectopic and molar pregnancy; O22.2 Superficial thrombophlebitis in pregnancy; O29.0 Complications of anesthesia during pregnancy; O42 Premature rupture of membranes; O70 Perineal laceration during delivery; O71 Other obstetric trauma; O74.0 Aspiration pneumonitis due to anesthesia during labor and delivery; O75 Other complications of labor and delivery, not elsewhere classified; O86.4 Pyrexia of unknown origin following delivery; O87.0 Superficial thrombophlebitis in the puerperium; O89.0 Pulmonary complications of anesthesia during the puerperium; O91 Infection of nipple associated with pregnancy, the puerperium and lactation; O98 Maternal infectious and

	parasitic diseases classifiable elsewhere but complicating; O99 Other maternal diseases classifiable elsewhere but complicating pregnancy, childbirth and the puerperium; P23.8 Congenital pneumonia due to other organisms; P23.9 Congenital pneumonia, unspecified; P24 Neonatal aspiration; P37.8 Other specified congenital infectious and parasitic diseases; P37.9 Congenital infectious or parasitic disease, unspecified; P39.4 Neonatal skin infection; P39.8 Other specified infections specific to the perinatal period; R57 Shock, not elsewhere classified; R65.0, R65.1, R65.9 Symptoms and signs specifically associated with systemic inflammation and infection, unspecified; R68.801 Toxaemia; R82.7, R83.5, R84.5, R85.5, R86.5, R87.5, R89.5 Abnormal microbiological findings of lab findings; S00 Superficial injury of head; Open wound of the body not classified as tier 1 (e.g., S01 Open wound of head; S11 Open wound of neck, et.); S05 Injury of eye and orbit (except S05.7); S20 Superficial injury of thorax; S60.902 Abrasion of hand; Burn and corrosion (except the first degree, e.g., T20.0 Burn of unspecified degree of head, face, and neck; T21.2 Burn of second degree of trunk, etc.); T33 Superficial frostbite; T34 Frostbite with tissue necrosis; T35 Frostbite involving multiple parts of the body and unspecified frostbite; T74.2 Sexual abuse, confirmed; T79.3 Wound infection after trauma, cannot be classified elsewhere; T81.1 Postprocedural shock; T81.4 Postprocedural shock; T81.5 Complications of foreign body accidentally left in body following procedure; T86.807 Infection after skin flap transplantation; T87.001 Infection following limb replantation; W44 Foreign bodies enter or penetrate the eye or natural cavity;W45 Foreign body or object entering through skin; W46 Contact with hypodermic needle; Unintentional cutting, acupuncture, perforation or bleeding during surgery, transfusion, dialysis, etc.(e.g., Y60.0 Unintentional cutting, acupuncture, perforation or bleeding during surgery); Failure of sterile precautions (e.g., Y62.000 Failure of sterile precautions during surgery); Z20 Contact with and (suspected) exposure to communicable diseases; Z22 Carrier of infectious disease (except carrier of virus).
**Tier 3 diagnoses that antibiotics are not indicated**	
Viral infections	A08 Viral and other specified intestinal infections; A60 Anogenital herpesviral [herpes simplex] infections; A63.0 Anogenital (venereal) warts; A80–A89 Viral and prion infections of the central nervous system; A90–A99 Arthropod–borne viral fevers and viral hemorrhagic fevers; B00–B09 Viral infections characterized by skin and mucous membrane lesions; B15–B19 Viral hepatitis; B20–B24 Human immunodeficiency virus [HIV] disease; B25–B34 Other viral diseases.
Fungal infections	B35–B49 Mycoses.
Non-infectious otitis media	H65 Nonsuppurative otitis media (except H65.101).
Viral upper respiratory infection (URI)	J00 Acute nasopharyngitis [common cold]; J04 Acute laryngitis and tracheitis; J05 Acute obstructive laryngitis [croup] and epiglottitis; J06 Acute upper respiratory infections of multiple and unspecified sites.
Influenza	J09 Influenza due to certain identified influenza viruses; J10 Influenza due to other

	identified influenza virus; J11 Influenza due to unidentified influenza virus. All of the above influenza except conditions with pneumonia.
Acute bronchitis	J20 Acute bronchitis; J21 Acute bronchiolitis.
Allergy and asthma	J45 Asthma; J30.1–J30.4 Allergic rhinitis; J39.3 Upper respiratory tract hypersensitivity reaction, site unspecified; Other allergy identified by key words in the diagnoses description, such as Z91.0 Allergy status, other than to drugs and biological substances, K52.2 Allergic and dietetic gastroenteritis and colitis, etc.
Non-infectious gastroenteritis	K29 Gastritis and duodenitis; K52 Other and unspecified noninfective gastroenteritis and colitis.
Non-specific symptoms, signs of respiratory system	R04 Hemorrhage from respiratory passages; R06 Abnormalities of breathing; R07 Pain in throat and chest; R09 Other symptoms and signs involving the circulatory and respiratory system.
Cough	R05 Cough.
Non-specific symptoms, signs of digestive system	R10 Abdominal and pelvic pain; R11 Nausea and vomiting; R12 Heartburn; R13 Aphagia and dysphagia; R14 Flatulence and related conditions; R15 Fecal incontinence; R16 Hepatomegaly and splenomegaly, not elsewhere classified; R17 Unspecified jaundice; R18 Ascites; R19 Other symptoms and signs involving the digestive system and abdomen.
Fever	R50 Fever of other and unknown origin.
Procedures and surgeries not included elsewhere	O04.9 Artificial abortion, complete or unspecified, without complications; Z98.8 Other specified postprocedural states and other surgeries and post-surgery situations (this was mainly identified by searching the key words that indicated the surgery).
All other conditions not listed above	All remaining diagnoses description not listed elsewhere, such as cancer, metabolic diseases, hypertension etc.

Table S3. Antibiotic prescription rates for various diagnosis categories in Chines primary care setting

	Urban		Rural		All regions	
	No. of visits (with antibiotics/overall)	Antibiotic prescription rate % (95% CI)	No. of visits (with antibiotics/overall)	Antibiotic prescription rate % (95% CI)	No. of visits (with antibiotics/overall)	Antibiotic prescription rate % (95% CI)
Pneumonia	7249/17824	40.7 (39.9–41.4)	15752/22201	71.0 (70.3–71.5)	23001/40025	57.5 (57.0–58.0)
Urinary tract infections	34667/63397	54.7 (54.3–55.1)	50049/67635	74.0 (73.7–74.3)	84716/131032	64.7 (64.4–64.9)
Certain bacterial diseases	2534/4867	52.1 (50.7–53.5)	1538/5230	29.4 (28.2–30.7)	4072/10097	40.3 (39.4–41.3)
Other bacterial infections	12799/67557	18.9 (18.7–19.2)	40438/122092	33.1 (32.9–33.4)	53237/189649	28.1 (27.9–28.3)
All tier 1 diagnoses	57249/153645	37.3 (37.0–37.5)	107777/217158	49.6 (49.4–49.8)	165026/370803	44.5 (44.3–44.7)
COPD	2935/11939	24.6 (23.8–25.4)	2502/4966	50.4 (49.0–51.8)	5437/16905	32.2 (31.5–32.9)
Acute sinusitis	810/1213	66.8 (64.0–69.4)	1513/2203	68.7 (66.7–70.6)	2323/3416	68.0 (66.4–69.6)
Acute pharyngitis	84910/162125	52.4 (52.1–52.6)	110926/152535	72.7 (72.5–72.9)	195836/314660	62.2 (62.1–62.4)
Acute otitis media	3800/4511	84.2 (83.1–85.3)	7203/7961	90.5 (89.8–91.1)	11003/12472	88.2 (87.6–88.8)
Other infectious diseases of the respiratory system	30744/87560	35.1 (34.8–35.4)	48344/77043	62.7 (62.4–63.1)	79088/164603	48.0 (47.8–48.3)
Infectious diseases of oral cavity and salivary glands	27729/50858	54.5 (54.1–55.0)	34987/63637	55.0 (54.6–55.4)	62716/114495	54.8 (54.5–55.1)
Infectious gastroenteritis	2071/3884	53.3 (51.7–54.9)	1953/3540	55.2 (53.5–56.8)	4024/7424	54.2 (53.1–55.3)
Other infectious diseases of the digestive system	2257/42577	5.3 (5.1–5.5)	6474/43722	14.8 (14.5–15.1)	8731/86299	10.1 (9.9–10.3)
Acne	18/638	2.8 (1.7–4.4)	118/1398	8.4 (7.0–10.0)	136/2036	6.7 (5.6–7.9)
Impetigo	15/108	13.9 (8.0–21.9)	5/15	33.3 (11.8–61.6)	20/123	16.3 (10.2–24.0)
Other skin, cutaneous, and mucosal infections	6700/14531	46.1 (45.3–46.9)	22462/31281	71.8 (71.3–72.3)	29162/45812	63.7 (63.2–64.1)
Other infectious diseases that antibiotic may be indicated	7917/42619	18.6 (18.2–18.9)	21989/87759	25.1 (24.8–25.3)	29906/130378	22.9 (22.7–23.2)
All tier 2 diagnoses	169906/422563	40.2 (40.1–40.4)	258476/476060	54.3 (54.2–54.4)	428382/898623	47.7 (47.6–47.8)

Viral infections	99/5369	1.8 (1.5–2.2)	1310/9340	14.0 (13.3–14.7)	1409/14709	9.6 (9.1–10.1)
Fungal infections	37/2334	1.6 (1.1–2.2)	82/5971	1.4 (1.1–1.7)	119/8305	1.4 (1.2–1.7)
Viral upper respiratory tract infection (URTI)	83599/672094	12.4 (12.4–12.5)	190289/524473	36.3 (36.2–36.4)	273888/1196567	22.9 (22.8–23.0)
Influenza	16/282	5.7 (3.3–9.1)	16/41	39.0 (24.2–55.5)	32/323	9.9 (6.9–13.7)
Acute bronchitis	45543/80351	56.7 (56.3–57.0)	61917/91504	67.7 (67.4–68.0)	107460/171855	62.5 (62.3–62.8)
Allergy and asthma	1162/47402	2.5 (2.3–2.6)	4964/40528	12.2 (11.9–12.6)	6126/87930	7.0 (6.8–7.1)
Cough	1862/32929	5.7 (5.4–5.9)	2975/15200	19.6 (18.9–20.2)	4837/48129	10.1 (9.8–10.3)
Other non-infectious gastroenteritis	5439/112337	4.8 (4.7–5.0)	20865/125952	16.6 (16.4–16.8)	26304/238289	11.0 (10.9–11.2)
Non-specific symptoms, signs of respiratory system	168/9261	1.8 (1.6-2.1)	1018/8334	12.2 (11.5-12.9)	1186/17595	6.7 (6.4-7.1)
Non-specific symptoms, signs of digestive system	968/18111	5.3 (5.0–5.7)	3258/21067	15.5 (15.0–16.0)	4226/39178	10.8 (10.5–11.1)
Fever	530/5605	9.5 (8.7–10.3)	1451/5611	25.9 (24.7–27.0)	1981/11216	17.7 (17.0–18.4)
Procedures and surgeries not included elsewhere	175/2354	7.4 (6.4–8.6)	687/5404	12.7 (11.8–13.6)	862/7758	11.1 (10.4–11.8)
All other conditions not listed above	92063/3743813	2.5 (2.4–2.5)	157155/1878772	8.4 (8.3–8.4)	249218/5622585	4.4 (4.4–4.4)
All tier 3 diagnoses	231661/4732242	4.9 (4.9–4.9)	445987/2732197	16.3 (16.3–16.4)	677648/7464439	9.1 (9.1–9.1)
Traditional Chinese medicine diagnoses	5415/858631	0.6 (0.6–0.6)	11001/454136	2.4 (2.4–2.5)	16416/1312767	1.3 (1.2–1.3)
Prescriptions not linked to any diagnosis	52/73714	0.1 (0.1–0.1)	154/72367	0.2 (0.2–0.2)	206/146081	0.1 (0.1–0.2)
All conditions	464283/6240795	7.4 (7.4–7.5)	823395/3951918	20.8 (20.8–20.9)	1287678/10192713	12.6 (12.6–12.7)

Table S4. Antibiotic prescription rates for various subgroups of patient visits in Chines primary care setting

		Urban			Urban			All areas	
	**No. of visits with antibiotics**	**Antibiotic prescription rate % (95% CI)**	**Standardized Antibiotic prescription rate % (95% CI)**	**No. of visits with antibiotics**	**Antibiotic prescription rate % (95% CI)**	**Standardized Antibiotic prescription rate % (95% CI)**	**No. of visits with antibiotics**	**Antibiotic prescription rate % (95% CI)**	**Standardized Antibiotic prescription rate % (95% CI)**
Overall	464283	7.4 (7.4–7.5)	8.8 (8.8–8.8)	823395	20.8 (20.8–20.9)	17.8 (17.8–17.9)	1287678	12.6 (12.6–12.7)	12.6 (12.6–12.7)
Type of primary care									
CHSCs/THs	140968	8.3 (8.3–8.3)	9.3 (9.2–9.3)	786942	21.4 (21.3–21.4)	18.4 (18.4–18.4)	927910	17.3 (17.2–17.3)	15.8 (15.7–15.8)
CHSSs/VCs	323315	7.1 (7.1–7.1)	8.9 (8.8–8.9)	36453	13.4 (13.3–13.5)	11.9 (11.8–12.1)	359768	7.5 (7.4–7.5)	9.0 (9.0–9.1)
Patient's age, years									
<6	27624	5.5 (5.5–5.6)	6.4 (6.2–6.6)	59394	26.9 (26.7–27.1)	19.0 (18.7–19.3)	87018	12.1 (12.0–12.2)	10.9 (10.7–11.1)
6–17	35078	11.3 (11.1–11.4)	10.2 (10.1–10.4)	96157	29.4 (29.2–29.6)	21.2 (21.0–21.3)	131235	20.5 (20.4–20.6)	16.0 (15.9–16.1)
18–44	130059	10.8 (10.7–10.8)	10.3 (10.3–10.4)	236284	21.6 (21.5–21.6)	18.3 (18.2–18.3)	366343	15.9 (15.8–15.9)	14.1 (14.0–14.1)
45–64	159543	7.0 (7.0–7.1)	9.3 (9.2–9.3)	283371	19.5 (19.4–19.5)	17.8 (17.7–17.8)	442914	11.9 (11.8–11.9)	12.9 (12.9–13.0)
≥65	111979	5.8 (5.7–5.8)	8.5 (8.4–8.5)	148189	17.4 (17.3–17.5)	16.8 (16.7–16.8)	260168	9.3 (9.3–9.3)	11.4 (11.4–11.5)
Patient's gender									
Male	217404	7.6 (7.6–7.7)	9.0 (9.0–9.0)	396039	21.6 (21.5–21.7)	18.3 (18.2–18.3)	613443	13.1 (13.1–13.1)	12.9 (12.9–13.0)
Female	246879	7.3 (7.3–7.3)	8.7 (8.7–8.7)	427356	20.2 (20.1–20.2)	17.5 (17.5–17.5)	674235	12.2 (12.2–12.3)	12.4 (12.3–12.4)
Payment type									
Insurance	396143	8.0 (8.0–8.0)	9.3 (9.2–9.3)	666344	21.5 (21.5–21.6)	18.1 (18.1–18.2)	1062487	13.2 (13.2–13.2)	13.0 (13.0–13.0)
Full out-of-pocket	68140	5.3 (5.3–5.4)	7.4 (7.3–7.4)	157051	18.3 (18.2–18.4)	17.3 (17.2–17.4)	225191	10.6 (10.5–10.6)	11.5 (11.5–11.6)
Year of visit									
2017	45946	9.2 (9.1–9.2)	9.1 (9.1–9.2)	131939	27.4 (27.2–27.5)	23.3 (23.2–23.5)	177885	18.1 (18.0–18.2)	16.2 (16.1–16.2)
2018	86622	8.3 (8.3–8.4)	8.8 (8.7–8.8)	236393	22.0 (22.0–22.1)	18.9 (18.8–18.9)	323015	15.3 (15.3–15.4)	14.1 (14.0–14.1)
2019	133886	8.1 (8.1–8.2)	8.7 (8.6–8.7)	211603	21.5 (21.4–21.6)	17.9 (17.8–18.0)	345489	13.1 (13.1–13.2)	12.3 (12.3–12.3)
2020	117142	6.5 (6.4–6.5)	8.7 (8.7–8.8)	159961	17.3 (17.2–17.4)	15.1 (15.0–15.1)	277103	10.1 (10.1–10.2)	11.3 (11.2–11.3)
2021	80687	6.5 (6.5–6.5)	9.5 (9.5–9.6)	83499	17.1 (17.0–17.2)	15.0 (15.0–15.1)	164186	9.5 (9.4–9.5)	11.4 (11.4–11.5)

Physician's age, years									
<30	21563	6.5 (6.4–6.6)	8.3 (8.2–8.4)	55432	19.0 (18.8–19.1)	16.2 (16.1–16.3)	76995	12.4 (12.3–12.4)	12.5 (12.4–12.5)
30–39	135743	6.3 (6.3–6.3)	8.0 (8.0–8.0)	342945	20.8 (20.7–20.9)	17.8 (17.8–17.9)	478688	12.6 (12.5–12.6)	12.7 (12.7–12.7)
40–49	132056	7.7 (7.7–7.7)	8.8 (8.8–8.9)	278515	22.6 (22.5–22.6)	18.9 (18.9–19.0)	410571	13.9 (13.9–14.0)	13.4 (13.4–13.4)
50–59	126229	9.2 (9.2–9.3)	9.8 (9.8–9.9)	117036	21.7 (21.6–21.8)	18.5 (18.4–18.6)	243265	12.8 (12.7–12.8)	12.4 (12.4–12.4)
≥60	48692	7.3 (7.2–7.3)	9.6 (9.5–9.7)	29467	12.4 (12.3–12.6)	12.8 (12.7–12.9)	78159	8.6 (8.6–8.7)	10.3 (10.2–10.3)
Physician gender									
Male	180119	8.0 (8.0–8.1)	9.7 (9.7–9.8)	504961	22.5 (22.5–22.6)	19.8 (19.8–19.9)	685080	15.3 (15.2–15.3)	14.9 (14.9–15.0)
Female	284164	7.1 (7.1–7.1)	8.3 (8.3–8.3)	318434	18.6 (18.6–18.7)	15.4 (15.3–15.4)	602598	10.6 (10.5–10.6)	10.8 (10.7–10.8)
Physician's education level									
Bachelor degree or above	104036	7.1 (7.1–7.1)	8.6 (8.6–8.7)	219668	21.3 (21.2–21.4)	19.2 (19.1–19.2)	323704	13.0 (12.9–13.0)	13.2 (13.2–13.3)
High school and below	360247	7.5 (7.5–7.6)	8.9 (8.8–8.9)	603727	20.7 (20.6–20.7)	17.4 (17.3–17.4)	963974	12.5 (12.5–12.6)	12.4 (12.4–12.4)

CHSCs: City community health service centers; THs: Township hospitals; CHSSs: community health service stations ; VCs: Village clinics.

Table S5. Sensitivity analyses for proportion of antibiotic prescriptions in each appropriateness category, all TCM diagnoses classified as tier 2 or tier 3.

	**Appropriate antibiotic use**		**Potentially appropriate antibiotic use**		**Inappropriate antibiotic use**		**Visits not linked to diagnosis**	
	**No. of prescriptions**	**Proportion, % (95% CI)**	**No. of prescriptions**	**Proportion, % (95% CI)**	**No. of prescriptions**	**Proportion, % (95% CI)**	**No. of prescriptions**	**Proportion, % (95% CI)**
TCM diagnoses classified by physicians	171056	13.3 (13.1–13.5)	463081	36.0 (35.8–36.1)	653335	50.7 (50.6–50.9)	206	0.02 (0.00–3.0)
All TCM diagnoses classified as tier 2	165026	12.8 (12.6–13.0)	496077	38.5 (38.4–38.7)	626369	48.6 (48.5–48.8)	206	0.02 (0.00–3.0)
All TCM diagnoses classified as tier 3	165026	12.8 (12.6–13.0)	428382	33.3 (33.1–33.4)	694064	53.9 (53.8–54.0)	206	0.02 (0.00–3.0)

Table S6. Standardized proportion of antibiotic prescriptions in each appropriateness category for various subgroups

	Appropriate antibiotic use, % (95% CI)	Potentially appropriate antibiotic use, % (95% CI)	Inappropriate antibiotic use, % (95% CI)	Visits not linked to diagnosis, % (95% CI)
Overall	13.3 (13.1–13.5)	36.0 (35.8–36.1)	50.7 (50.6–50.9)	0.02 (0.00–3.0)
Area type				
Urban	14.9 (14.5–15.2)	43.4 (43.1–43.7)	41.7 (41.4–42.0)	0.01 (0.00–10.7)
Rural	10.9 (10.7–11.2)	29.5 (29.3–29.8)	59.5 (59.3–59.7)	0.02 (0.00–4.2)
Type of primary care				
CHSCs/THs	11.8 (11.5–12.0)	31.9 (31.7–32.1)	56.3 (56.2–56.5)	0.02 (0.00–3.5)
CHSSs/VCs	14.3 (13.9–14.7)	42.5 (42.2–42.8)	43.2 (42.9–43.5)	0.01 (0.00–15.4)
Patient's age, years				
<6	19.1 (18.3–19.9)	31.3 (30.6–32.0)	49.6 (49.0–50.2)	0.01 (0.00–32.5)
6–17	13.1 (12.4–13.7)	28.8 (28.2–29.3)	58.2 (57.7–58.6)	0.03 (0.00–14.1)
18–44	12.9 (12.5–13.2)	32.5 (32.2–32.9)	54.6 (54.3–54.9)	0.01 (0.00–11.7)
45–64	13.6 (13.2–13.9)	36.5 (36.2–36.8)	50.0 (49.7–50.2)	0.01 (0.00–8.7)
≥65	14.0 (13.5–14.4)	40.4 (40.0–40.8)	45.6 (45.3–46.0)	0.01 (0.00–14.4)
Patient's gender				
Male	13.3 (13.0–13.6)	35.4 (35.1–35.7)	51.3 (51.1–51.5)	0.01 (0.00–6.6)
Female	13.5 (13.2–13.7)	36.4 (36.1–36.6)	50.1 (49.9–50.3)	0.02 (0.00–5.1)
Payment type				
Insurance	12.8 (12.6–13.0)	36.7 (36.5–36.9)	50.5 (50.3–50.7)	0.02 (0.00–3.6)
Full out-of-pocket	15.3 (14.9–15.8)	33.6 (33.1–34.0)	51.1 (50.7–51.4)	0.02 (0.00–14.6)
Year of visit				
2017	9.7 (9.1–10.2)	28.6 (28.1–29.1)	61.7 (61.3–62.1)	0.01 (0.00–30.2)
2018	11.6 (11.2–12.0)	32.6 (32.2–33.0)	55.8 (55.5–56.1)	0.01 (0.00–14.0)
2019	13.2 (12.8–13.6)	36.6 (36.3–36.9)	50.2 (49.9–50.5)	0.02 (0.00–9.5)
2020	15.6 (15.1–16.0)	40.3 (40.0–40.7)	44.1 (43.7–44.4)	0.03 (0.00–7.0)
2021	15.9 (15.3–16.5)	40.4 (39.9–40.9)	43.7 (43.2–44.2)	0.01 (0.00–35.8)
Physician's age, years				
<30	13.8 (13.0–14.6)	33.9 (33.1–34.6)	52.3 (51.7–53.0)	0.01 (0.00–51.5)
30–39	13.9 (13.6–14.3)	37.0 (36.7–37.3)	49.1 (48.8–49.3)	0.02 (0.00–5.2)
40–49	12.3 (11.9–12.6)	34.5 (34.2–34.8)	53.2 (53.0–53.5)	0.01 (0.00–12.5)
50–59	13.2 (12.8–13.7)	35.8 (35.4–36.2)	51.0 (50.6–51.4)	0.01 (0.00–17.3)
≥60	14.2 (13.4–15.0)	41.1 (40.4–41.8)	44.7 (44.0–45.3)	0.01 (0.00–40.3)
Physician gender				
Male	12.2 (12.0–12.5)	33.1 (32.9–33.4)	54.6 (54.4–54.8)	0.02 (0.00–4.4)
Female	14.6 (14.3–14.9)	38.9 (38.6–39.1)	46.5 (46.2–46.7)	0.01 (0.00–9.7)
Physician's education level				

Bachelor degree or above	14.3 (13.9–14.7)	36.7 (36.3–37.0)	49.0 (48.7–49.3)	0.03 (0.00–6.7)
High school and below	13.1 (12.8–13.3)	35.8 (35.6–36.1)	51.1 (50.9–51.3)	0.01 (0.00–5.4)

CHSCs: City community health service centers; CHSSs: community health service stations; THs: Township hospitals; VCs: Village clinics.

Table S7. Most commonly prescribed antibiotics for different diagnosis categories

	Appropriate antibiotic prescribing	Potentially appropriate antibiotic prescribing	Inappropriate antibiotic prescribing	TCM diagnoses	Not linked to any diagnosis	All visits
**Antibacterial spectrum**						
Broad	154122 (80.9)	350707 (72.8)	569245 (78.5)	12615 (73.2)	941 (78.9)	1087630 (76.8)
Narrow	36342 (19.1)	131022 (27.2)	156245 (21.5)	4629 (26.8)	252 (21.1)	328490 (23.2)
**Individual antibiotics**						
Cefuroxime	17927 (9.4)	83098 (17.2)	140859 (19.4)	1675 (9.7)	152 (12.7)	243711 (17.2)
Amoxicillin	12500 (6.6)	78113 (16.2)	125474 (17.3)	2682 (15.6)	91 (7.6)	218860 (15.5)
Levofloxacin	72411 (38.0)	42216 (8.8)	71569 (9.9)	2534 (14.7)	195 (16.3)	188925 (13.3)
Cefalexin	7909 (4.2)	51922 (10.8)	97207 (13.4)	2135 (12.4)	59 (4.9)	159232 (11.2)
Cefixime	8094 (4.2)	52529 (10.9)	76698 (10.6)	1438 (8.3)	162 (13.6)	138921 (9.8)
Amoxicillin-clavulanate	4318 (2.3)	25722 (5.3)	28336 (3.9)	1254 (7.3)	79 (6.6)	59709 (4.2)
Ceftriaxone	7494 (3.9)	18994 (3.9)	27281 (3.8)	294 (1.7)	109 (9.1)	54172 (3.8)
Azithromycin	2340 (1.2)	15277 (3.2)	35504 (4.9)	520 (3.0)	37 (3.1)	53678 (3.8)
Metronidazole	8252 (4.3)	30124 (6.3)	11962 (1.6)	449 (2.6)	15 (1.3)	50802 (3.6)
Norfloxacin	21961 (11.5)	5259 (1.1)	20309 (2.8)	1060 (6.1)	29 (2.4)	48618 (3.4)
Ornidazole	13748 (7.2)	20601 (4.3)	5759 (0.8)	463 (2.7)	80 (6.7)	40651 (2.9)
Clindamycin	2214 (1.2)	10835 (2.2)	22922 (3.2)	411 (2.4)	13 (1.1)	36395 (2.6)
Cefradine	517 (0.3)	5555 (1.2)	10120 (1.4)	481 (2.8)	3 (0.3)	16676 (1.2)
Ceftezole	1344 (0.7)	6540 (1.4)	5634 (0.8)	5 (0.0)	65 (5.4)	13588 (1.0)
Cefazolin	724 (0.4)	4595 (1.0)	6730 (0.9)	54 (0.3)	7 (0.6)	12110 (0.9)
Gentamicin	816 (0.4)	2315 (0.5)	7161 (1.0)	303 (1.8)	9 (0.8)	10604 (0.7)
Roxithromycin	403 (0.2)	4165 (0.9)	4503 (0.6)	170 (1.0)	11 (0.9)	9252 (0.7)
Ceftizoxime	1278 (0.7)	4488 (0.9)	3283 (0.5)	73 (0.4)	57 (4.8)	9179 (0.6)
Others	6214 (3.3)	19381 (4.0)	24179 (3.3)	1243 (7.2)	20 (1.7)	51037 (3.6)

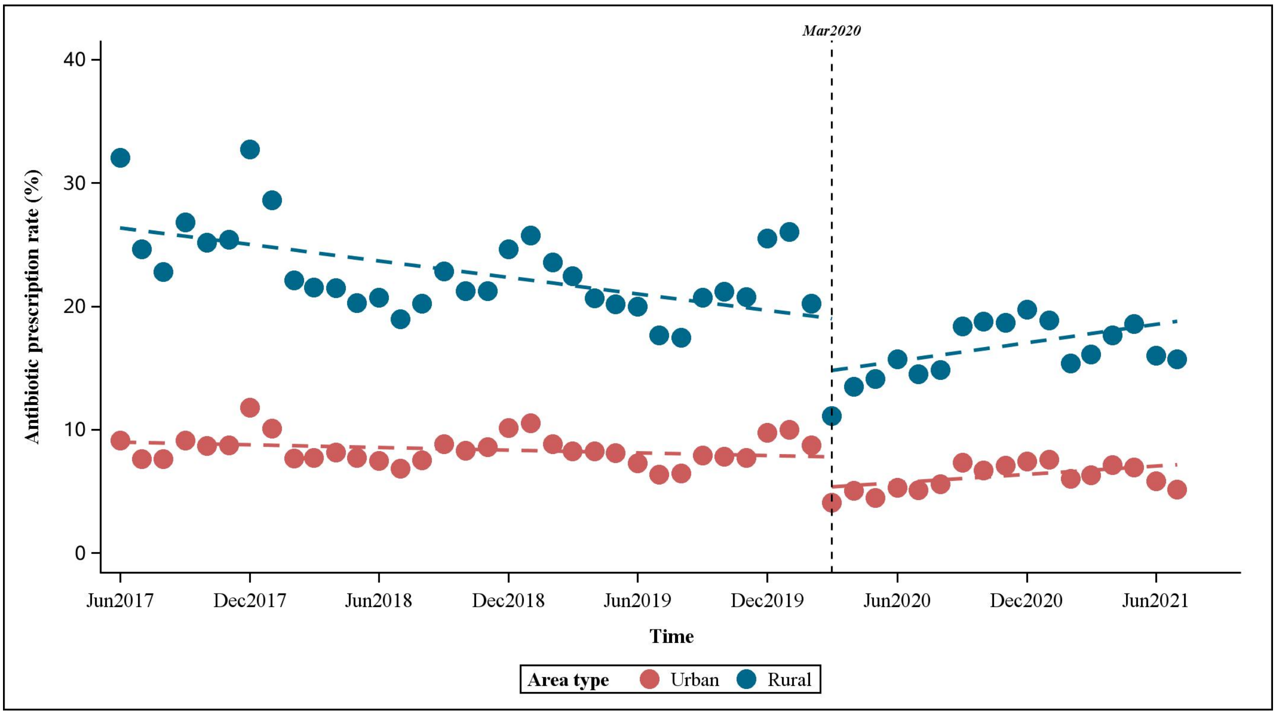

Figure S1. Impact of COVID-19 on the antibiotic prescription rate in the urban and rural primary care setting.

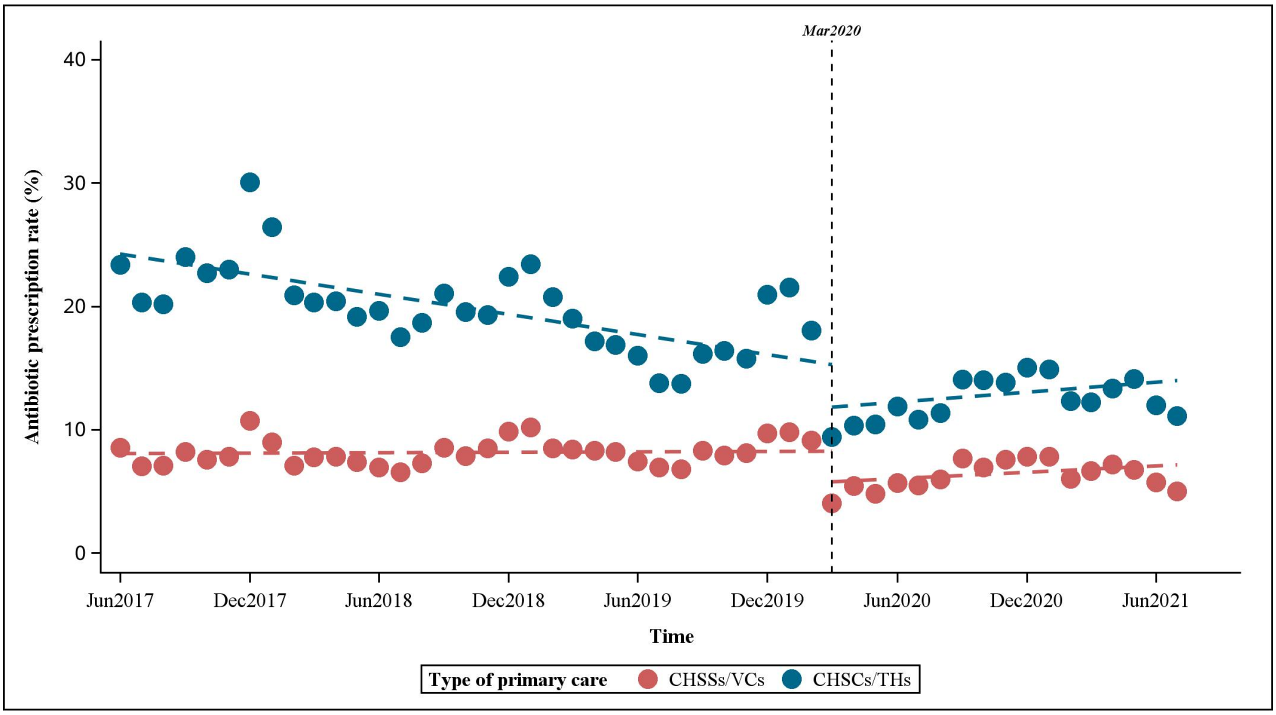

Figure S2. Impact of COVID-19 on the antibiotic prescription rate in the CHSCs/THs and CHSSs/VCs.

CHSCs: City community health service centers; CHSSs: community health service stations; THs: Township hospitals; VCs: Village clinics.

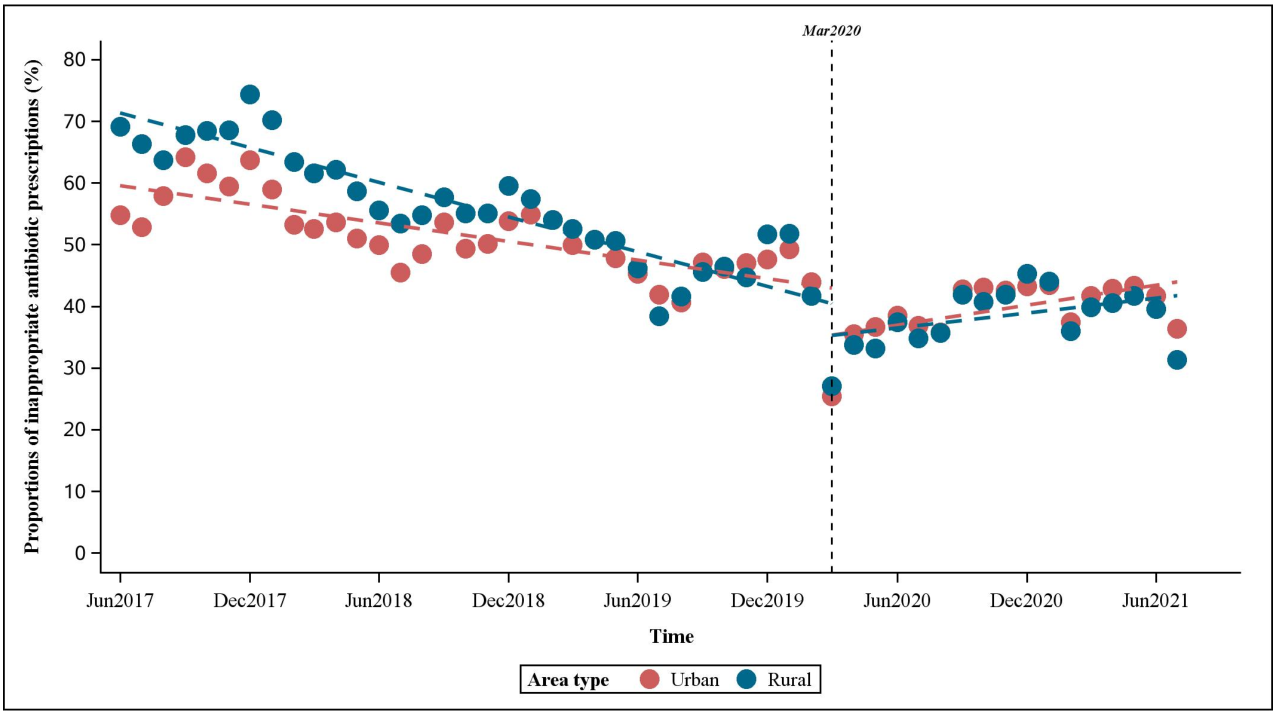

Figure S3. Impact of COVID-19 on the proportion of inappropriate antibiotic prescriptions in the urban and rural primary care setting.

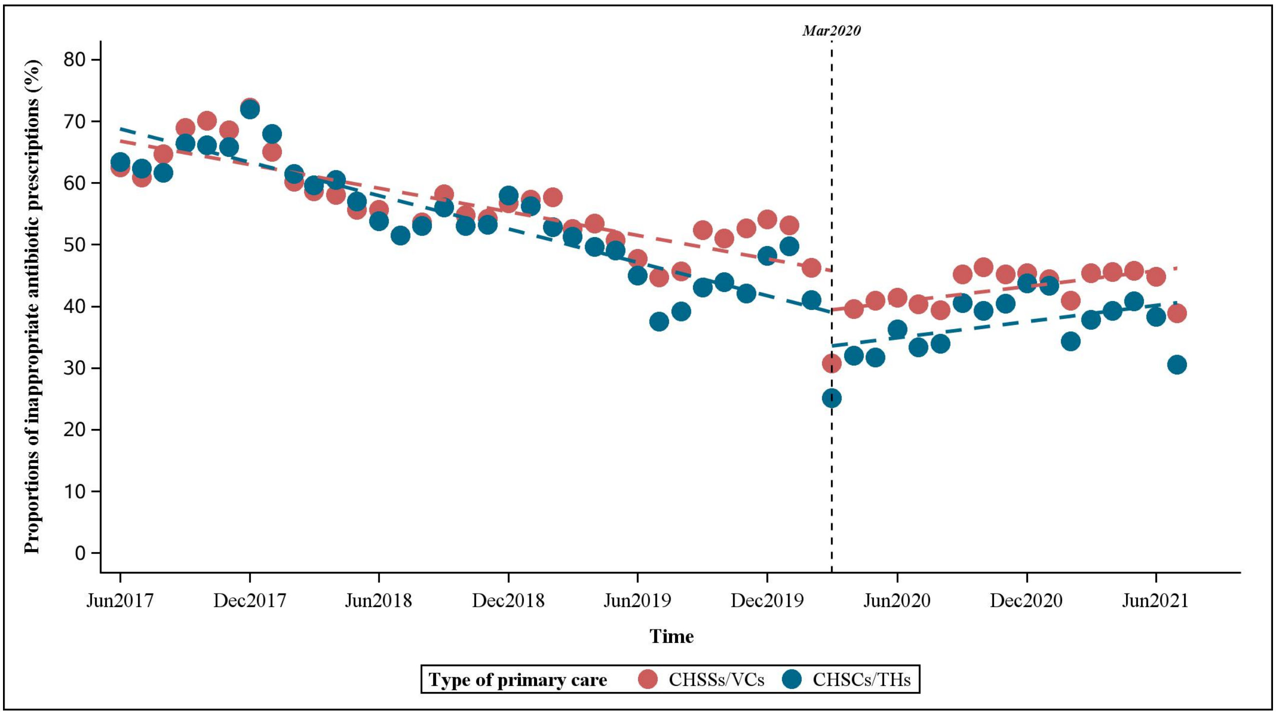

Figure S4. Impact of COVID-19 on the proportion of inappropriate antibiotic prescriptions in the CHSCs/THs and CHSSs/VCs.

CHSCs: City community health service centers; CHSSs: community health service stations; THs: Township hospitals; VCs: Village clinics.

Table S8. Impact of COVID-19 on the antibiotic prescription rates in visits for different diagnosis categories.

	Trend before COVID-19	Level change	Slope change	Trend after COVID-19
Tier 1 diagnoses	0.2 (0.1 – 0.3)	-2.2 (-4.2 – -0.2)	0.2 (0.10 – 0.3)	0.4 (0.3 – 0.5)
Tier 2 diagnoses	-0.06 (-0.1 – 0.00)	-5.0 (-6.7 – -3.3)	0.5 (0.3 – 0.6)	0.4 (0.3 – 0.6)
Tier 3 diagnoses	-0.3 (-0.3 – -0.2)	-2.1 (-3.6 – -0.6)	0.4 (0.3 – 0.5)	0.1 (0.00 – 0.2)
Traditional Chinese medicine diagnoses	-0.08 (-0.1 – -0.05)	0.2 (-0.3 – 0.7)	0.07 (0.04 – 0.1)	-0.01 (-0.02 – -0.01)

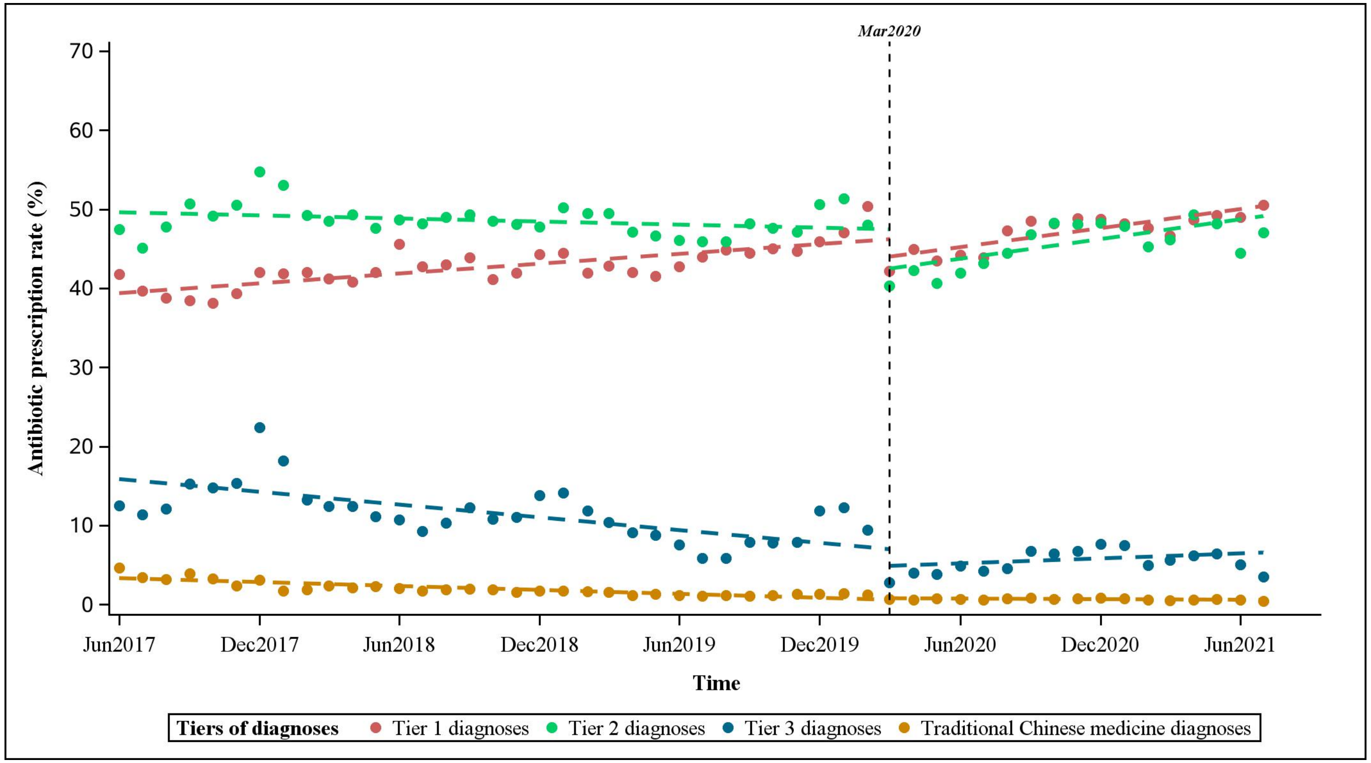

Figure A5. Impact of COVID-19 on the antibiotic prescription rates in visits for different diagnosis categories.

References

1. Zhao H, Bian J, Wei L, et al. Validation of an algorithm to evaluate the appropriateness of outpatient antibiotic prescribing using big data of Chinese diagnosis text. *BMJ Open.* 2020;10(3):e31191.

2. Zhao H, Wei L, Li H, et al. Appropriateness of antibiotic prescriptions in ambulatory care in China: a nationwide descriptive database study. *Lancet Infect Dis.* 2021;21(6):847-857.
